# Thr^4^ phosphorylation on RNA Pol II occurs at early transcription regulating 3′-end processing

**DOI:** 10.1126/sciadv.adq0350

**Published:** 2024-09-06

**Authors:** Rosamaria Y. Moreno, Svetlana B. Panina, Seema Irani, Haley A. Hardtke, Renee Stephenson, Brendan M. Floyd, Edward M. Marcotte, Qian Zhang, Y. Jessie Zhang

**Affiliations:** Department of Molecular Biosciences, University of Texas, Austin, TX, USA.

## Abstract

RNA polymerase II relies on a repetitive sequence domain (YSPTSPS) within its largest subunit to orchestrate transcription. While phosphorylation on serine-2/serine-5 of the carboxyl-terminal heptad repeats is well established, threonine-4’s role remains enigmatic. Paradoxically, threonine-4 phosphorylation was only detected after transcription end sites despite functionally implicated in pausing, elongation, termination, and messenger RNA processing. Our investigation revealed that threonine-4 phosphorylation detection was obstructed by flanking serine-5 phosphorylation at the onset of transcription, which can be removed selectively. Subsequent proteomic analyses identified many proteins recruited to transcription via threonine-4 phosphorylation, which previously were attributed to serine-2. Loss of threonine-4 phosphorylation greatly reduces serine-2 phosphorylation, revealing a cross-talk between the two marks. Last, the function analysis of the threonine-4 phosphorylation highlighted its role in alternative 3′-end processing within pro-proliferative genes. Our findings unveil the true genomic location of this evolutionarily conserved phosphorylation mark and prompt a reassessment of functional assignments of the carboxyl-terminal domain.

## Teaser

Thr^4^ phosphorylation starts in early transcription, recruits transcription regulators, and is crucial for 3′-end processing.

## INTRODUCTION

The intricate functions of eukaryotic cells depend on the transcription activities of RNA polymerases I, II, and III. Among these, RNA polymerase II (Pol II) stands out as the primary workhorse, responsible for transcribing all messenger RNAs (mRNAs) for protein expression as well as some small nuclear RNA (snRNA) and small nucleolar RNA (snoRNA) (*1*–*3*). To effectively manage the substantial workload, Pol II features a unique C-terminal domain (CTD) in its largest subunit RPB1. This domain is characterized by a conserved repetitive sequence of seven residues YSPTSPS (repeated 26 times for *Saccharomyces cerevisiae* and 52 for *Homo sapiens*) and is crucial for coordinating mRNA production and processing (*4*). While occasional deviations from the consensus sequence occur (mostly in the seventh position and sometimes in the fourth), the overarching presence of the repetitive heptad sequence remains consistent across eukaryotes (*5*). The CTD does not affect the catalytic activity of RNA Pol II, but its absence or even replacement of specific residues can result in cell death (*6*, *7*).

The distinctive repetitive sequence of the CTD has garnered considerable attention in efforts to comprehend its role in eukaryotic transcription. Central to the CTD’s functionality is its capacity for phosphorylation. Five of the seven residues undergo phosphorylation, and blocking this process results in the cessation of transcription (*8*). Notably, Ser^5^ and Ser^2^ are believed to get phosphorylated at specific stages of transcription—initiation and elongation/termination, respectively (*9*, *10*). These phosphorylation events recruit key transcriptional regulatory proteins to the transcribing Pol II to facilitate transcription with precision (*11*). Conversely, the roles of the other three phosphorylatable residues of the consensus heptad (Tyr^1^, Thr^4^, and Ser^7^) are less clearly defined with limited knowledge about their cellular function, despite confirmation of their phosphorylation in cells. Thr^4^, in particular, remains the most mysterious.

**Fig. 1.**
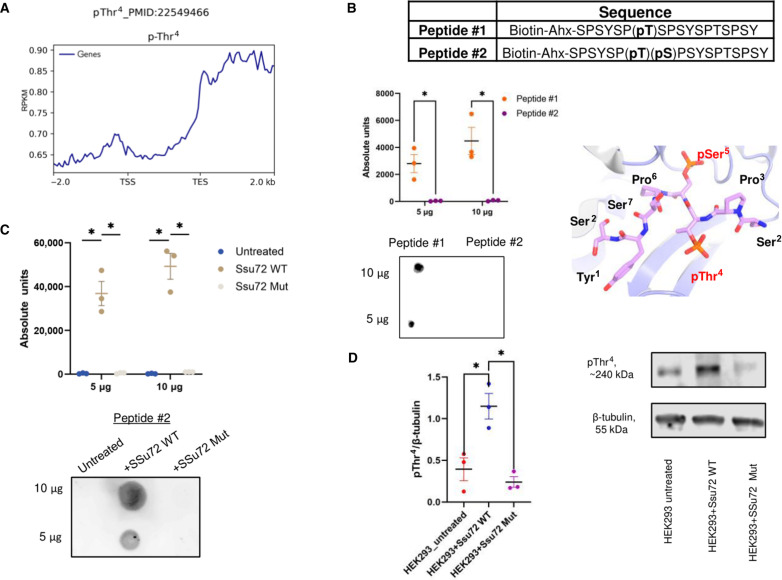
pThr^4^ antibody signal is perturbed by neighboring pSer^5^ and enhanced by Ssu72 treatment. (**A**) ChIP profile of pThr^4^ Pol II dataset from PMID: 22549466. The region between the TSS and TES is scaled to 2000 bp for every gene; −2 kb corresponds to −2 kb from the TSS; +2 kb corresponds to +2 kb from the TES. (**B**) Dot blot loaded with 5 or 10 μg of CTD peptide #1 (singly phosphorylated pT4) or CTD peptide #2 (doubly phosphorylated pT4pS5) and incubated with pThr^4^ (6D7) antibody. Structure of Ssu72 bound to a pSer^5^ CTD for specific targeting of pSer^5^ dephosphorylation (PDB: 4IMJ) is shown. Comparison was performed using unpaired *t* test. (**C**) Dot blot showing serial dilutions of pT4S5 CTD peptide treated with WT Ssu72/symplekin or catalytically deficient Ssu72 C13D D144N/symplekin and incubated with the pThr^4^ antibody. Comparisons were performed using unpaired *t* tests. (**D**) WB showing pThr^4^ recognition in HEK293 cell lysate treated with Ssu72 or mutant Ssu72 lacking activity. Comparison was performed using unpaired (Ssu72 WT versus untreated) or paired (Ssu72 WT vs Ssu72 Mut) *t* tests. In all plots, means with SEM are shown and quantification was from three independent biological replicates. *P < 0.05.

Genetic studies using Thr^4^ variants identified elongation and termination defects when phosphorylation at this position is disrupted as well as implications for Thr^4^ in mitotic cell cycle regulation (*12*–*15*). Despite these putative Thr^4^ functions, previous chromatin immunoprecipitation sequencing (ChIP-seq) indicates that Thr^4^ phosphorylation does not occur until late in transcription, reaching its maximum around 500 to 2000 bases after the polyadenylation site (Fig. 1A) (*16*). Although multiple lines of evidence from both genetic and chemical perturbance studies implicate Thr^4^ in elongation and coprocessing of mRNA, the absence of Thr^4^ phosphorylation enrichment in ChIP profiles during the early stages of transcription seems to contradict its proposed functions. Furthermore, a seemingly conservative mutation of Thr^4^ replaced with alanine in the Pol II CTD, results in cell death in human cells but not in *S. cerevisiae* or *Schizosaccharomyces pombe* (*16*–*18*). Thus, the role of Thr^4^ and its phosphorylation in eukaryotic transcription are still very puzzling.

ChIP is a powerful tool for unraveling the role of transcriptional regulators, but its efficacy heavily relies on the antibodies used in the experiments. While antibodies targeting the phospho-specific Thr^4^ of CTD exhibit high specificity, avoiding cross-recognition with other phospho-CTD epitopes, their ability to recognize pThr^4^ is susceptible to interference from flanking phosphorylation marks on a hyperphosphorylated Pol II (*16*). Specifically, neighboring Ser^5^ and Ser^2^ phosphorylation can impede recognition of the pThr^4^ epitope when tested on a heptad polypeptide (*16*). This phenomenon, known as the “masking” effect, introduces the risk of false-negative signals and ambiguity in the genome-wide distribution analysis of pThr^4^.

Here, we leverage the exceptional specificity of a well-characterized CTD phosphatase to remove the masking effect for pThr^4^ in situ, which allows us to delineate the genomic location of pThr^4^ and embark on an exploration of pThr^4^ function. Notably, because Ser^5^ phosphorylation occurs promptly upon transcription initiation, the potential masking of pThr^4^ by pSer^5^ raises valid concerns of underestimating the level of Thr^4^ phosphorylation and the challenge of accurately pinpointing its genomic location. Intriguingly, our investigation uncovered a distinct peak immediately following the transcription start site (TSS), accounting for almost 50% of pThr^4^ peaks without masking events. Intriguingly, protein-coding genes exhibit a different pThr^4^ profile from noncoding RNA genes. In protein-coding genes, Thr^4^ phosphorylation level drops significantly after the TSS peak, but it starts to rise to a plateau close to the transcription end site (TES), which is missing in noncoding genes. A proteomic study using a reconstructed phospho-CTD system unveiled a noteworthy overlap of approximately two-thirds of the pThr^4^ and pSer^2^ CTD interactomes. Notably, this overlap was particularly pronounced among proteins harboring a CTD binding motif known as the CTD-interacting domain (CID). Subsequent x-ray crystallographic examination of a representative protein, RPRD1B, shed light on the fact that CIDs recognize the CTD via either Ser^2^ or Thr^4^ phosphorylation in an analogous manner that is conserved across all CIDs. The ChIP analyses of such proteins are consistent with their recruitment via pThr^4^ rather than pSer^2^. Our mechanistic study reveals that Thr^4^ phosphorylation primes the transcription effect of Ser^2^ phosphorylation. Functional studies focused on a Thr^4^ variant incapable of phosphorylation unearthed a pivotal role for Thr^4^ in productive Pol II elongation and termination at the proper polyadenylation site, critical for the stability of mRNA.

## RESULTS

### Masking effects of flanking phosphorylation on pThr^4^ detection in human cells

Previous studies that profiled pThr^4^ Pol II on the human genome revealed most recruitment occurs after the TES (Fig. 1A) (*16*). This unexpected genomic location raised concerns over the accuracy of this profile because the same report indicates that Thr^4^ antibody recognition may be blocked by nearby Ser^2^ or Ser^5^ phosphorylation on CTD (*16*). This concern is particularly significant in human cells as Thr^4^-Ser^5^ double phosphorylation accounts for ~20% of double phosphorylation on heptads, as reported in an in-depth analysis of endogenous human CTD phosphorylation mapping using mass spectrometry (MS) (*19*).

Because ChIP experiments heavily rely on antibody specificity, we first tested if the pThr^4^ antibody (6D7) cross-recognizes other CTD phosphorylation sites. Using peptides of 18-nucleotide oligomer (about 2.5 heptad repeats) phosphorylated at different sites, we observed no detection of any other phosphorylation sites by the pThr^4^ antibody, confirming its high specificity (fig. S1A). Next, we evaluated the masking effect of Ser^5^ phosphorylation on pThr^4^, a frequent double phosphorylation detected in human cells (Fig. 1B). We used synthetic peptides of 18-nucleotide oligomers with pThr^4^pSer^5^ double phosphorylation (Fig. 1B). Strong detection of pThr^4^ was observed in the singly phosphorylated peptide (peptide #1), but no signal was detected when a pSer^5^ followed the pThr^4^ (peptide #2) at the same peptide concentration. Thus, neighboring pSer^5^ blocks detections of pThr^4^ by the pThr^4^ antibody. When transcription occurs in cells, phosphorylation of Ser^5^ occurs at the beginning of transcription after preinitiation complex (PIC) assembles (*9*). Thus, if the Thr^4^ were phosphorylated, then it would likely not be detected by the pThr^4^ antibody.

Another reported Thr^4^ antibody masking effect in vitro is observed when Ser^2^, two residues upstream of pThr^4^, is phosphorylated (**pSer**^**2**^Pro^3^**pThr**^**4**^Ser^5^). Despite the in vitro blocking effect, this potential impediment posed to pThr^4^ identification is less concerning under physiological conditions as pSer^2^/pThr^4^ double phosphorylation is rather rare in cells (*19*). Subsequent biochemical and cellular experiments have shown that, although Ser^2^ and Thr^4^ phosphorylations occur around the same time during transcription cycle, they tend to occur on different heptads because kinases avoid placing a phosphate on the neighboring Ser^2^ when Thr^4^ is phosphorylated and vice versa (*19*). When we used a Ser^2^ kinase, Dyrk1a, to phosphorylate an 18-nucleotide oligomer synthetic peptide containing phosphorylated Thr^4^ (fig. S1B), pThr^4^ detection was not affected, probably due to the Ser^2^ on different heptads was favored for phosphorylation (fig. S1C).

Another phosphorylation mark co-occurring with Thr^4^ on the same heptad is Tyr1 (accounts for ~5% of doubly phosphorylated heptads) (*19*). Previous experiments using synthetic peptides with pTyr1/pThr^4^ double phosphorylation did not report a masking effect (*16*). To corroborate this, we evaluated the possibility that Tyr1 phosphorylation affects pThr^4^ detection by treating pThr^4^-containing CTD peptide with the Tyr1 kinase, c-Abl (fig. S1B). We found that c-Abl treatment does not affect pThr^4^ antibody recognition (fig. S1C). Last, the antibody characterization showed that Ser^7^ on the same heptad repeat does not affect epitope recognition for Thr^4^ phosphorylation (*16*). Thus, the pThr^4^ antibody 6D7 is highly specific for pThr^4^ but might fail to recognize pThr^4^ when it co-occurs with Ser^5^ phosphorylation in cells.

### Removing masking effect of Ser^5^ phosphorylation on pThr^4^ ChIP

The reported enrichment of pThr^4^pSer^5^ double phosphorylation in cells raised concerns for Thr^4^ phosphorylation detection as the masking effect by adjacent phosphorylation may block pThr^4^ recognition leading to false-negative results (*19*). To address this issue, we explored the possibility of eliminating the masking effect on pThr^4^ antibody recognition by selectively removing the interfering phosphate groups on Ser^5^ of the CTD. Our prior biochemical and structural analyses have established phosphatase Ssu72 as a highly specific phosphatase for Ser^5^ of the CTD, distinguishing Ser^5^ from any other Ser/Thr residues in the CTD heptad (*20*–*22*). Our earlier MS and biochemical assays affirm that Ssu72 exerts no effect on the levels of pSer^2^ or pThr^4^ while effectively eradicating Ser^5^ phosphorylation on the CTD (*21*). The structural element that establishes such high specificity is attributed to the requirement that Pro^3^ must be in the cis configuration to fit into the Ssu72 active site (Fig. 1B) (*21*). Phospho-Ser^5^ extends into the active center of Ssu72 when the heptad forms a tight β turn (Fig. 1B). This β turn is sterically hindered by the Tyr1 residue of the same heptad preventing pSer^2^ or pThr^4^ placed into the active site (fig. S1D). In addition, the dephosphorylation of Ser^5^ by Ssu72 is not blocked by flanking phosphorylation, for example, on pThr^4^ (Fig. 1B) (*20*). Biochemically, Ssu72 exhibits weak activity against pSer^7^, about three magnitudes lower than the activity exhibited toward pSer^5^ (*23*). This “star activity,” however, is inconsequential for our purpose of profiling pThr^4^ in the physiological context because the Ser^7^ phosphorylation state does not affect Thr^4^ antibody recognition. Thus, the masking effect in Thr^4^ ChIP profiling, stemming from the flanking Ser^5^ phosphorylation, can potentially be removed using Ssu72 as a biochemical tool.

We first tested experimentally if the selective removal of Ser^5^ phosphorylation by Ssu72 will enhance the detection of pThr^4^ on a pThr^4^pSer^5^ doubly phosphorylated peptide. Ssu72 exhibits maximum stability and activity when it is associated with a scaffolding protein called symplekin (*24*). Therefore, we purified the Ssu72/symplekin complex and treated the pThr^4^pSer^5^ doubly phosphorylated peptide (peptide #2) (Fig. 1C). Our results indicate that the recognition of pThr^4^ on the doubly phosphorylated peptide by 6D7, which was previously undetectable before phosphatase treatment, exhibited a strong signal upon Ssu72/symplekin treatment (Fig. 1C). To ensure that the observed effect is due to dephosphorylation by Ssu72 rather than other interfering factors, we conducted the same experiment with a catalytically inactive mutant, Ssu72 C13D/D144N. In the absence of the phosphatase activity, 6D7 is unable to recognize pThr^4^ (Fig. 1C).

We then investigated if the Ssu72/symplekin phosphatase complex could enhance the recognition of pThr^4^ in the cell lysate by removing pSer^5^ in the nuclear cell extract. We obtained the nuclear extract from human embryonic kidney (HEK) 293 cells and treated it with no phosphatase, active Ssu72/symplekin [wild-type (WT)], or catalytically inactive Ssu72/symplekin phosphatase complex (C13D/D144N variant of Ssu72). Immunoblotting with the pThr^4^ antibody 6D7 revealed an approximately threefold increase in signal intensity for the phosphatase-treated sample compared to the untreated and the catalytically dead phosphatase-treated samples (Fig. 1D). This suggests that there is a large percentage of Thr^4^ being masked. However, this is different for the pSer^2^ signal in the nuclear cell extract treated with Ssu72 and detected by the pSer^2^ antibody 3E10, showing no significant difference in recognition compared to untreated and inactive Ssu72 mutant samples (fig. S1E). Thus, the treatment of a hyperphosphorylated Pol II CTD using the CTD phosphatase Ssu72 allows for the detection of pThr^4^ that was previously blocked by flanking Ser^5^ phosphorylation.

### ChIP-seq analysis of pThr^4^ of the CTD

**Fig. 2.**
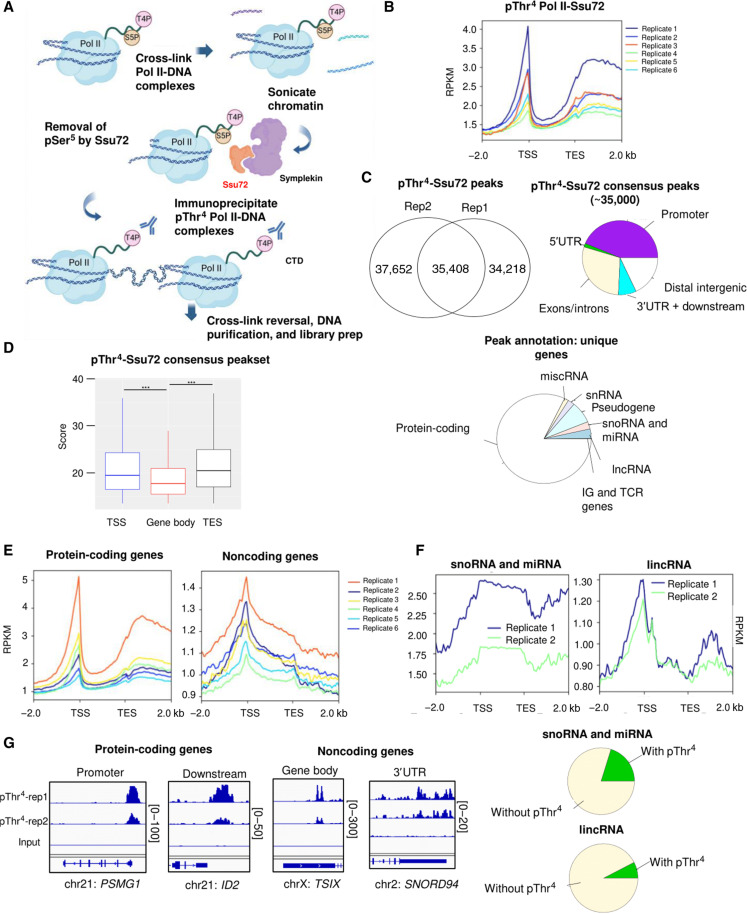
Unbiased mapping of Thr^4^ phosphorylation on the human genome. (**A**) Workflow of pThr^4^ ChIP experiments with Ssu72 phosphatase treatment. (**B**) ChIP-seq profile of pThr^4^ Pol II with Ssu72 treatment along human annotated genes. The region between the TSS and TES is scaled to 2000 bp for every gene; −2 kb corresponds to −2 kb from the TSS; +2 kb corresponds to +2 kb from the TES. (**C**) Left pie graph shows the number of consensus pThr^4^ Pol II ChIP-seq peaks between two replicates of Ssu72-treated samples. Right pie graph shows the distribution of genomic locations of consensus peaks between two replicates of pThr^4^-Ssu72. Promoter region is defined as (−1 kb; +1 kb) from the TSS. Bottom pie graph splits consensus peaks into protein-coding or noncoding gene categories. (**D**) Box plot of scores’ distribution over the TSS, gene body, and the TES for consensus pThr^4^ peaks. Promoter region is defined as (−1 kb; +1 kb) from the TSS. Outliers not shown. Groups were compared using Kruskal-Wallis tests with post hoc pairwise tests. ***P < 0.001. (**E**) Normalized ChIP-seq profile of pThr^4^ Pol II along protein-coding or noncoding genes. (**F**) pThr^4^ signal distribution over snoRNA/miRNA genes or lincRNA (RPKM). Pie graphs (below) denote the localization of pThr^4^ Pol II within genomic coordinates of snoRNA/miRNA or lincRNA genes. (**G**) IGV tracks showing the pThr^4^ signal on several protein-coding or noncoding genes.

To map pThr^4^ Pol II phospho-marks over the human genome using ChIP-seq, we used phosphatases to selectively remove the masking pSer^5^ and expose pThr^4^ for antibody binding. After optimizing the time and amount of phosphatase treatment (fig. S2, A and B), we performed ChIP-seq by adding the Ssu72/symplekin complex after the cell lysis step (Fig. 2A). The nuclear lysate was incubated with the phosphatase solution at 4°C for 30 min before binding to the 6D7 antibody (Fig. 2A). To enhance the rigor of our study, we prepared six biological replicates for the ChIP analysis and assessed the reproducibility of pThr^4^ ChIP-seq signal. The higher-than-normal sampling was to interrogate how different factors such as cell amount, cell density, and treatment variation affect the outcome of the profile. All the six performed replicates had appropriate immunoprecipitation quality estimated by fraction of reads in peaks (FriP) scores (≥4%), consistent with 1% threshold in the ENCODE guidelines (fig. S2C) (*25*). As another ChIP-seq quality control (QC), several select genomic loci with high estimated pThr^4^ enrichment were validated using ChIP–quantitative polymerase chain reaction (qPCR) under Ssu72 treatment versus control (fig. S2D).

The mapping of all the six datasets over the human genome produced the same profile for pThr^4^—a sharp peak at the TSS followed by a relatively low level of pThr^4^ until the signal increases to form a plateau steadily at the TES (Fig. 2B). Our data show that the reads per kilobase per million mapped reads (RPKM) values at the TSS peak is consistently higher than maximal RPKM values at the TES plateau (TSS/TES ratios ranged from 1.01 to 1.27). We also performed pThr^4^ ChIP without Ssu72 treatment and found a significant difference in the TSS/TES ratio wherein the peak at the TSS is markedly lower compared to replicate datasets with Ssu72 treatment (fig. S2E). Previously, it has been noticed that 5% of the genes have a very small peak at the TSS in pThr^4^ ChIP (*16*). Our enriched pThr^4^ binding at the TSS can explain a potential role in transcription elongation where T4A mutation of CTD prevents Pol II from proceeding after the TSS (*16*). Because Ser^5^ phosphorylation is mostly enriched at the TSS, it is likely that removing its masking effect reveals a high abundance of pThr^4^. Thus, the Thr^4^ phosphorylation profile highlights a sharp peak at the beginning of the transcription. We also notice the plateau near the TES seems to be slightly shifted compared to previous pThr^4^ ChIP (*16*), yet the transcriptional implication of such shift requires a follow-up study.

Of the six analyzed ChIP-seq datasets, replicates 1 and 2 had the highest FriP scores (10 and 6%, respectively) (fig. S2C) and the highest numbers of MACS2-called broad pThr^4^ peaks (*q* < 0.1; 71,235 and 77,353 peaks, respectively) (table S1). Therefore, we focused on replicates 1 and 2 for more detailed follow-up analysis and derived a consensus pThr^4^ peakset shared between them (Fig. 2C, left). First, analysis of the genomic distribution of the consensus pThr^4^ peaks revealed that nearly half of the peaks were located at the promoters, defined as (−1 kb; +1 kb) from the TSS (Fig. 2C, right). The absolute majority (>75%) of pThr^4^ peaks were mapped to protein-coding genes (Fig. 2C, bottom). Furthermore, pThr^4^ peaks located in promoters and the TES had significantly higher scores (*P* < 2.2 × 10^−16^) than peaks mapped to the gene body (exons/introns) (Fig. 2D), highlighting the biological significance of the pThr^4^ signal in both transcription start and end. Consistently, the T4A mutant of Pol II was reported to have significantly deregulated, pausing index both at the 5′-initiation site (increased stalling) and 3′-termination site (decreased stalling) compared to WT control (*16*).

One of the first questions we addressed was if all the genes shared the same pThr^4^ profile because it was reported that pThr^4^ has a special function in snoRNA termination (*26*). In addition, we noticed weak pThr^4^ peaks in snoRNA, microRNA (miRNA), and long noncoding RNA (lncRNA) genes (Fig. 2C). To answer that, we separated the genes into protein-coding versus noncoding sequences (Fig. 2E). The analysis revealed that the pThr^4^ profile over protein-coding genes was similar to the overall profile with the sharp peak at the TSS and plateau at the TES region (Fig. 2E). In contrast, noncoding RNAs had a markedly different profile with a wide peak around the TSS region (Fig. 2E). Despite the difference in profile, the score distribution of peaks across snoRNA/miRNA genes followed the same pattern (fig. S2F). To identify how abundant pThr^4^ binding was in noncoding RNAs, we downloaded genomic regions corresponding to “snoRNA/miRNA” and “lincRNA transcripts” tracks (hg19) from the UCSC Table Browser and overlapped them with genomic coordinates of the consensus pThr^4^ peakset. A total of 459/2273 unique snoRNA and miRNA sequences (20%) and 1643/21,630 (~8%) long intergenic noncoding RNA (lincRNA) sequences had consensus pThr^4^ peaks (Fig. 2F) (*26*). Figure 2G shows examples of pThr^4^ peaks in protein-coding and noncoding genes. Overall, ChIP-seq results suggest that the distribution of pThr^4^ binding appears to follow a gene-specific pattern and maps predominantly to TSS and TES regions of protein-coding genes.

### Pol II CTD pThr^4^ interactomes

**Fig. 3.**
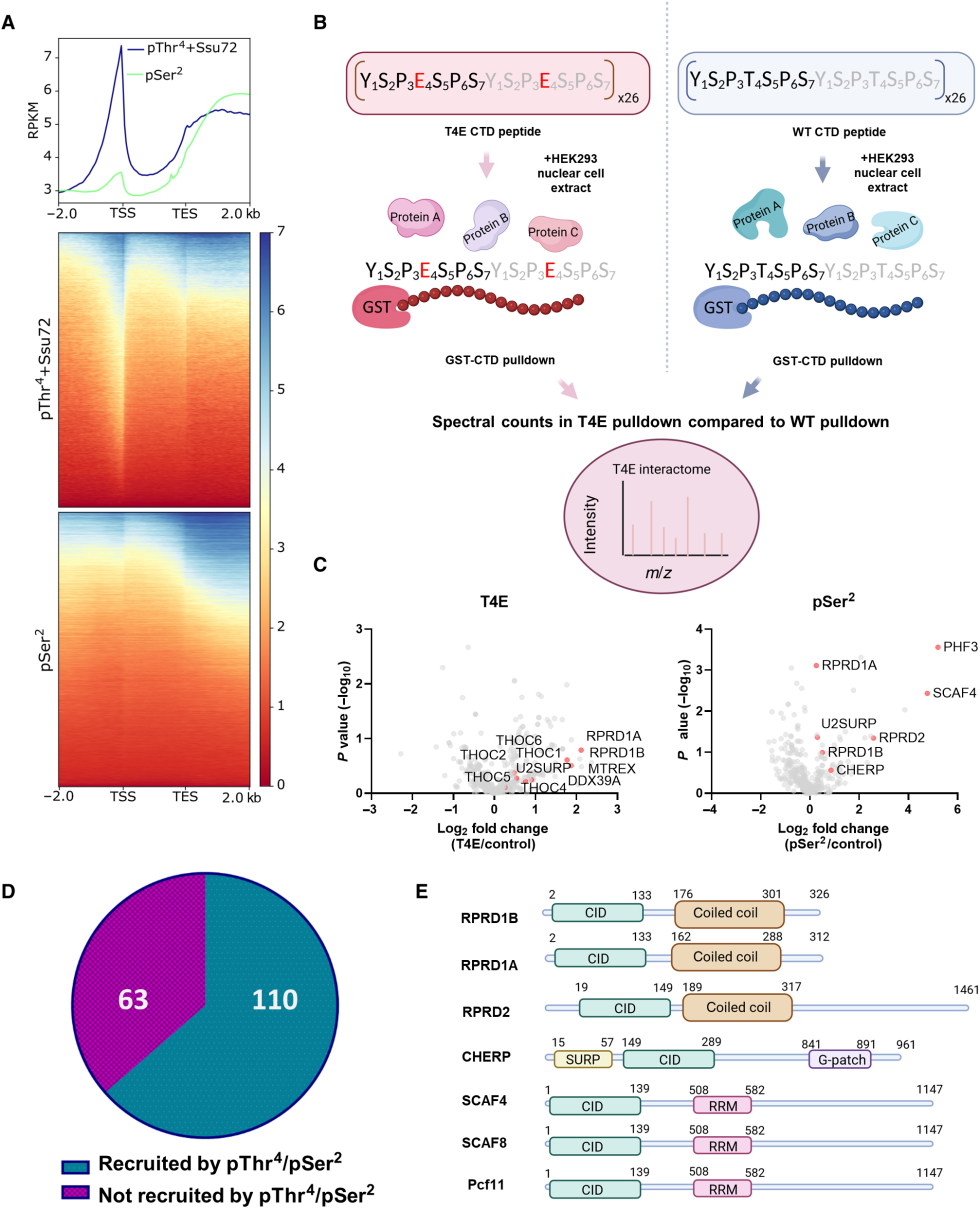
Recruitment pattern of Thr^4^ phosphorylation. (**A**) Normalized ChIP signal of pThr^4^ Pol II (samples were treated with Ssu72) and pSer^2^ Pol II along human annotated genes. The region between the TSS and TES is scaled to 2000 bp for every gene; −2 kb corresponds to −2 kb from the TSS; +2 kb corresponds to +2 kb from the TES. (**B**) Schematic showing the workflow of T4E pulldown. A 26X CTD peptide with Thr^4^ mutated to glutamate in every repeat was used as a bait and compared to unphosphorylated WT 26X CTD. Phosphorylated CTD substrates were incubated with the HEK293 nuclear cell extract overnight. CTD substrates were pulled down using glutathione beads and MS/MS analysis was conducted. (**C**) Volcano plots comparing T4E or pSer^2^ immunoprecipitation to unphosphorylated CTD immunoprecipitation as a control. Enriched factors were determined using a *P* value of <0.05. Factors mentioned in text are labeled and shown as red dots. (**D**) Pie graph showing proteins present in both T4E and pSer^2^ immunoprecipitation that are either positively or negatively enriched in both pulldowns have differing recruitment compared to control. (**E**) Schematic showing the protein domain architecture of human CID proteins.

The latter half of the pThr^4^ ChIP profile where it plateaus close to the TES is reminiscent of the previous mapping of Ser^2^ phosphorylation across various cell types and organisms, highlighting a tendency for these two phosphorylation marks to accumulate and plateau near the TES (*4*). To compare the genomic localization of pThr^4^ and pSer^2^, we performed ChIP-seq against pSer^2^ in the HEK293 cell line (Fig. 3A). The profiles of pThr^4^ and pSer^2^ exhibit similarity in shape and transcriptional timing, although pSer^2^ has only weak signals at the TSS (Fig. 3A). Although the different antibodies used in the pSer^2^ and pThr^4^ ChIP do not allow for a direct quantifiable comparison, the profiles do imply that pThr^4^ and pSer^2^ occurrence during transcription might be coinciding.

To elucidate the function of Thr^4^ phosphorylation in eukaryotic transcription, we conducted a proteomic analysis to identify proteins recruited by pThr^4^ and compared it to proteins recruited in a pSer^2^ pulldown, considering the highly similar genomic location of the two CTD posttranslational modifications (PTMs) (Fig. 3C). We recombinantly expressed a 26X GST-CTD construct with Thr^4^ mutated to glutamate at every heptad to mimic the negative charge of a phosphoryl group. Using a label-free proteomic approach, we conducted pulldowns using a GST-26X T4E-CTD as the “bait” protein with an unphosphorylated GST-26X CTD as a control (Fig. 3B). Equal amounts of the nuclear cell lysate containing phosphatase/protease inhibitors were added to individual samples (fig. S3A) and incubated overnight while mixing. After several salt washes, the samples were analyzed by comparing the abundance of pulled-down proteins in phosphorylated samples compared to that in the control.

In parallel, we conducted pSer^2^ pulldown using a protocol we described before with in vitro reconstruction of pSer^2^ phosphorylation (*27*). Comparison of the pSer^2^ interactome and the T4E pulldown revealed a large proportion of proteins that appeared concurrently in both the T4E and pSer^2^ samples (Fig. 3C and table S2). Notably, 110 proteins (64% of the total) demonstrated simultaneous recruitment or depletion between the two pulldowns, underscoring the overlapping recruitment profiles of pSer^2^ and pThr^4^ (Fig. 3D and fig. S3, B and C).

A close inspection of the proteins pulled down by the pSer^2^ or pThr^4^-mimic (T4E) reveals an overrepresentation of proteins containing a protein motif called the CID (Fig. 3E and fig. S3D). This binding motif is highly conserved throughout eukaryotes, and most identified CIDs exhibit selective binding toward pSer^2^ over pSer^5^. In our proteomic study, proteins containing this domain were spotted in at least one if not both pulldowns, including RPRD1A, RPRD1B, RPRD2, SCAF4, and U2SURP (Fig. 3E). This parallel result led us to wonder if, generally, CIDs are dually capable of recognizing either pSer^2^ or pThr^4^ in vitro. Almost all known CID-containing proteins have been implicated in termination and mRNA processing. A previous detailed characterization of yeast Rtt103 (which contains a CID) has shown that it can bind phosphorylated Thr^4^ in addition to binding phosphorylated Ser^2^ (*28*). SCAF4/SCAF8 are CID proteins that suppress early, alternative polyadenylation (APA) sites and regulates transcriptional termination (*29*). Similarly, Rtt103, a yeast CID termination factor, forms a complex with Rat1/Rai1 (*30*) and is important in regulating termination of both protein-coding and a subset of noncoding genes through interactions with pThr^4^ marks on snoRNA (*13*). Furthermore, a Cleavage and Polyadenylation (CPA) complex member, PCF11, is highly prevalent at the 3′ end of genes (*31*) and influences APA site usage, with decreased PCF11 levels leading to isoforms with distal sites chosen (*32*). The role of CID proteins in termination might be linked to pThr^4^ phosphorylation rather than pSer^2^ phosphorylation.

### CID motif–containing protein RPRD1B can bind both pSer^2^ and pThr^4^

**Fig. 4.**
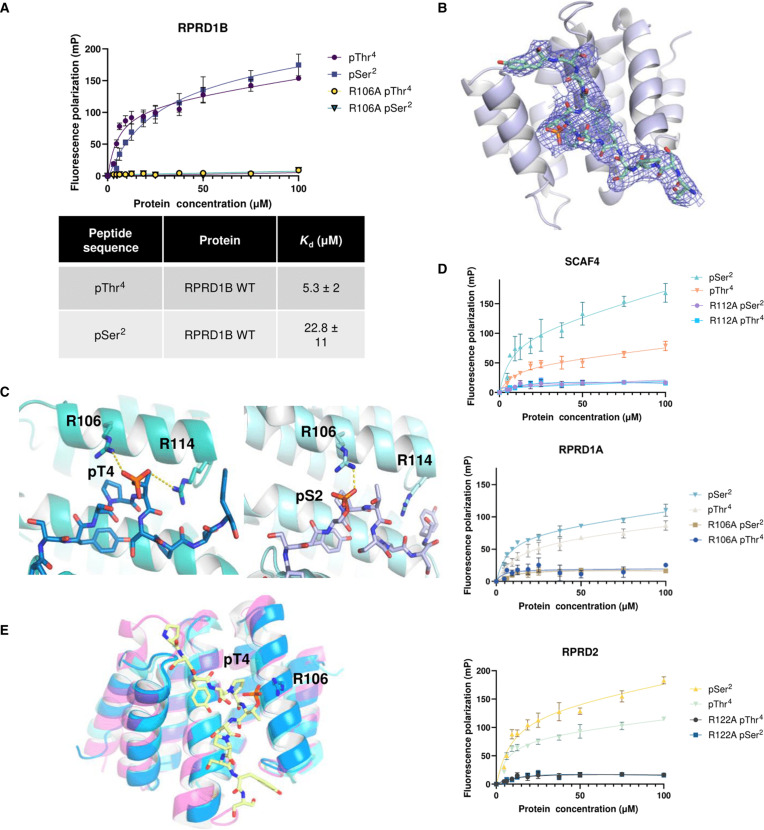
CID proteins recognize both pSer^2^ and pThr^4^ CTD. (**A**) FA measurements of the CID of RPRD1B with FITC- labeled pS2/pT4 CTD peptides. (**B**) 2*F*_o_-*F*_c_ electron density map (contour to 1.0 s) of RPRD1B’s CID complexed with the pThr^4^ CTD peptide. (**C**) Side-by-side view of RPRD1B binding to the pThr^4^ or pSer^2^ CTD peptide (PDB: 4Q94). Conserved Arg^106^ and Arg^114^ are shown with stick representation. (**D**) FA measurements of WT or mutant CID of SCAF4, RPRD1A, and RPRD2 with pS2/pT4 CTD peptides. Experimental isotherms were fitted to a total binding model. Binding assays were performed in triplicate. Error bars indicate the SD. (**E**) Structural modeling of the conserved recognition of CID of SCAF4 (pink), RPRD1A (blue), and RPRD2 (light blue) binding to the pThr^4^ CTD peptide.

To test if proteins containing a CID motif that were previously characterized as pSer^2^ binders can also bind to pThr^4^, we started with a structural and biophysical analysis of RPRD1B, a human transcription regulator that contains a CID that strongly interacts with pSer^2^. We first used fluorescence anisotropy (FA) to measure the interaction of RPRD1B with a 16-nucleotide oligomer CTD polypeptide phosphorylated at a single position either at Ser^2^ or at Thr^4^ located in the middle of the heptad. Purified RPRD1B can bind to both pSer^2^ and pThr^4^ CTD peptides, with a little tighter association to the pThr^4^ peptide [dissociation constant (*K*_d_) of 22.8 ± 11 μM for pSer^2^ and 5.3 ± 2 μM for pThr^4^] (Fig. 4A). To investigate the recognition mode of human CID proteins to a pThr^4^ CTD, we cocrystallized RPRD1B with a 15-nucleotide oligomer, singly phosphorylated Thr^4^ CTD peptide. We determined the complex structure at 2.5 Å (statistics for data collection and refinement in table S6). The RPRD1B-CID is composed of eight α helices arranged in a right-handed superhelical array, characteristic of the conserved CID fold (*33*–*35*). RPRD1B exists as a monomer in our crystal structure, consistent with its oligomerization state in solution as shown in its gel filtration profile (fig. S4A) (*35*). We observed a stretch of elongated positive density, consistent with the CTD polypeptide based on which we modeled in 12 residues of the 15-nucleotide oligomer CTD peptide (Fig. 4B and fig. S4B). Previously, RPRD1B has been cocrystallized with a CTD peptide containing Ser^2^ phosphorylation [Protein Data Bank (PDB): 4Q94] (*35*). Unexpectedly, the mode of binding in the CTD backbone is almost identical in the two structures, with the CTD peptide anchored in place in both structures through several hydrophobic pockets (fig. S4C). The aromatic ring of Tyr^1^ of the CTD is situated in a hydrophobic pocket formed by Val^23^ and Tyr^61^, and Pro^3^ fits into a hydrophobic core composed of Tyr^61^, Leu^104^, Leu^107^, and Ile^110^ (fig. S4C). The hydroxyl of Tyr1 forms hydrogen bonds with Asp^65^ (fig. S4C). Notably, in both structures, the recognition of the phospho-CTD residue depends on Arg^106^ (Fig. 4C). Arg^106^ forms salt bridge interactions with the phosphate group when the phosphate group is extended either from Ser^2^ or Thr^4^ (Fig. 4C). Mutations in this arginine residue do not alter the stability of RPRD1B (fig. S4D) but do abolish binding to pThr^4^ or pSer^2^ (Fig. 4A). The only difference in the CTD peptide recognition of RPRD1B for pSer^2^ or pThr^4^ CTD peptide is R114, which can potentially form a salt bridge with the phosphate group of the Thr^4^ phosphate but is located too far (6.9 Å) from the Ser^2^ phosphate (Fig. 4C).

### CID is a dual-binding module

Our structure of RPRD1B in complex with pThr^4^ CTD peptides indicates that Arg^106^ mediates pSer^2^/pThr^4^ recognition through salt bridge formation to the phosphorylated CTD residue (Fig. 4A). This arginine is conserved across all CID proteins except for Nrd1 in *S. cerevisiae*, which does not exhibit significant binding toward pSer^2^. We thus speculated that CIDs might be all capable of binding to pThr^4^ and pSer^2^ via this conserved arginine. A pSer^2^ binding protein we just identified, CHERP, can also bind to a pThr^4^ CTD (*K*_d_ = 10.7 ± 2 μM for pSer^2^ and 3.9 ± 1 μM for pThr^4^) (*27*). To verify the potential dual recognition of pSer^2^ and pThr^4^ by CID-containing proteins, we isolated the CIDs of RPRD1A, RPRD2, and SCAF4 and quantified their binding to CTD phospho-peptides using FA (fig. S4, E and F). Similar to RPRD1B and CHERP, RPRD1A, RPRD2, and SCAF4 all exhibit strong binding to both pSer^2^ and pThr^4^ CTD peptides with comparable affinity (RPRD1A, *K*_d_ = 12.3 ± 5 μM for pThr^4^ and 13.8 ± 1 μM for pSer^2^; RPRD2, *K*_d_ = 6.6 ± 2 μM for pThr^4^ and 8.7 ± 2 μM for pSer^2^; and SCAF4, *K*_d_ = 8.1 ± 4 μM for pThr^4^ and 6.7 ± 4 μM) (Fig. 4D). The structures of these CIDs are highly similar to RPRD1B, with all key residues for CTD recognition conserved (Fig. 4E). Structural superimposition reveals that RPRD1A, RPRD2, and SCAF4 share an identical binding groove as RPRD1B. Notably, the position of the conserved Arg that can bind the CTD phosphate group can extend and interact with either Ser^2^ or Thr^4^ (Fig. 4E). Thus, transcription regulators containing a CID, previously identified as Ser^2^ binding proteins, can also bind to pThr^4^, sometimes with an even stronger affinity than that observed for pSer^2^.

### RNA Pol II recruits CID-containing proteins through pThr^4^/pSer^2^

**Fig. 5.**
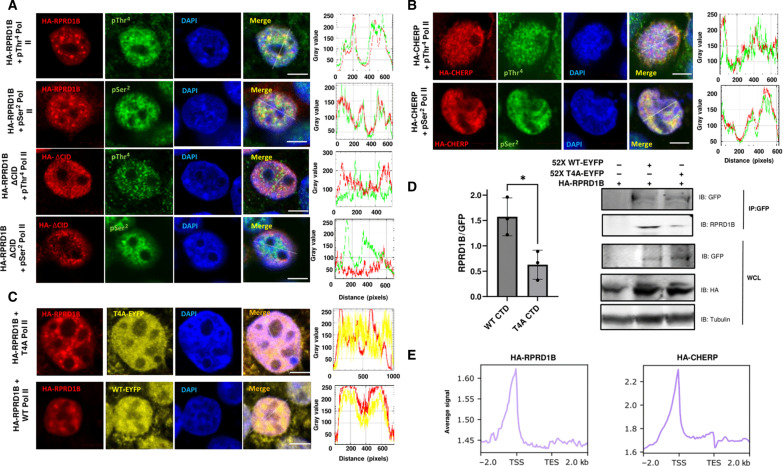
CID proteins colocalize with pSer^2^ and pThr^4^ Pol II. (**A**) Representative confocal fluorescent images of HA-RPRD1B full length or HA-RPRD1B ΔCID (red), pSer^2^ or pThr^4^ Pol II (green), and DAPI (blue) in HEK293 cells. Scale bars, 5 μm. Profile intensity plots between red and green channels are shown. (**B**) Confocal fluorescent images of HA-CHERP full length (red) and pSer^2^/pThr^4^ Pol II (green). Scale bars, 5 μm. (**C**) Confocal fluorescent images of HA-RPRD1B (red) and 52X T4A Pol II or 52X WT Pol II (yellow). Scale bars, 5 μm. Profile intensity plots between red and green or red and yellow channels are shown. All IF experiments were performed three independent times. (**D**) Anti-YFP coimmunoprecipitation of T4A Pol II or WT Pol II with HA-RPRD1B. Representative blots are shown, and quantification is based on three independent biological replicates. Plot shows means with SD. IB, immunoblot; IP, immunoprecipitation; WCL, whole-cell lysate. (**E**) Distribution of the input normalized ChIP signal of HA-RPRD1B or HA-CHERP across human annotated genes (*27*). Average peak signal between a window 2 kb upstream/downstream from the TSS/TES. *P < 0.05.

The in vitro association of CID-containing proteins with the phospho-CTD motivates us to evaluate their association with RNA Pol II in cells. Using RPRD1B as an example, we first examined the cellular location through immunofluorescence (IF) staining in HEK293 cells (Fig. 5A). As anticipated, HA-RPRD1B and pSer^2^ Pol II colocalize in transfected HEK293 cells. IF of HA-RPRD1B and pThr^4^ Pol II reveals substantial overlap in Pol II clusters (Fig. 5A). However, colocalization of RPRD1B with a hyperphosphorylated Pol II is greatly diminished when RPRD1B lacks a CID (Fig. 5A). The marked drop in colocalization indicates that RPRD1B relies on the CID interaction to associate with a phosphorylated Pol II at either Thr^4^ or Ser^2^ during ongoing transcription, echoing our in vitro observations of RPRD1B interacting with phospho-CTD polypeptides.

Recently, through proteomic studies, we identified a transcription splicing factor, CHERP, as a pSer^2^ binding protein (*27*). Similar to RPRD1B and other pSer^2^ binding CID proteins, CHERP also exhibits strong binding to pThr4 CTD in vitro (*27*). We thus explore if CHERP colocalizes with a Thr^4^ phosphorylated RNA Pol II in cells. When we conducted the same IF assay, hemagglutinin (HA)–tagged CHERP colocalizes extensively with phosphorylated Ser^2^ and Thr^4^ Pol II (Fig. 5B). As we showed previously, the recruitment of CHERP to RNA Pol II relies on its CID (*27*). Thus, the CIDs found in transcription regulators, which mediate binding to pSer^2^ and pThr^4^ in vitro, are responsible for the recruitment of their parent proteins to ongoing transcription.

To understand the function of T4 phosphorylation, we generated a construct of mammalian RPB1 with a CTD in which Thr^4^ in every heptad repeat in the 52 repeats of RPB1 is replaced by alanine, thus impossible to get phosphorylated at the Thr^4^ position. To test if the recruitment of RPRD1B relies on Thr^4^ phosphorylation, we visualized the subcellular localization of HA-RPRD1B in conjunction with either RPB1 T4A-EYFP or WT-EYFP (Fig. 5C). We observed colocalization of RPRD1B with WT RPB1 but not in the T4A variant. We next examined if RPRD1B associates with RPB1 T4A in the cell extract. To this end, we performed coimmunoprecipitation assays with extracts from cells expressing similar protein levels of RPB1 WT-EYFP or T4A-EYFP. Using the enhanced yellow fluorescent protein (EYFP) tag for immunoprecipitation, we found that endogenous RPRD1B coimmunoprecipitates with WT RPB1, but there is a significant reduction in binding toward RPB1 T4A (Fig. 5D).

To identify the genomic location for CID-containing proteins, we conducted ChIP-seq analysis of HA-RPRD1B. We expressed an HA-tagged full-length RPRD1B in HEK293 cells and used double cross-linking for ChIP-seq analysis as RPRD1B binding to chromatin is an indirect interaction through direct Pol II binding (fig. S5A). The ChIP profile of RPRD1B shows a high peak around the TSS with a small flat plateau close to the TES of genes. The profile is consistent between biological replicates with high reproducibility (Fig. 5E and fig. S5, B to D). RPRD1B has been detected at the downstream region of the *LEO1* gene (*36*) as well as showing strong occupancy at the promoter and polyadenylate [poly(A)] cleavage sites of the cyclin D1 gene (*37*). The profile for genomic binding of RPRD1B is reminiscent of the result in our CHERP study where a similar profile is found with a pronounced peak right at the TSS of the genes (Fig. 5E). Notably, the genomic localization of CHERP was eliminated when the CID was omitted in CHERP (*27*). In both cases, the genomic location of these CID-containing proteins resembles the pThr^4^ ChIP profile more than the pSer^2^ ChIP profile. Together, all our data suggest that there is a strong likelihood that CID-containing transcription regulators are recruited to the transcription apparatus through Thr^4^ phosphorylation rather than Ser^2^ phosphorylation.

### T4 phosphorylation cross-talks with S2 phosphorylation

**Fig. 6.**
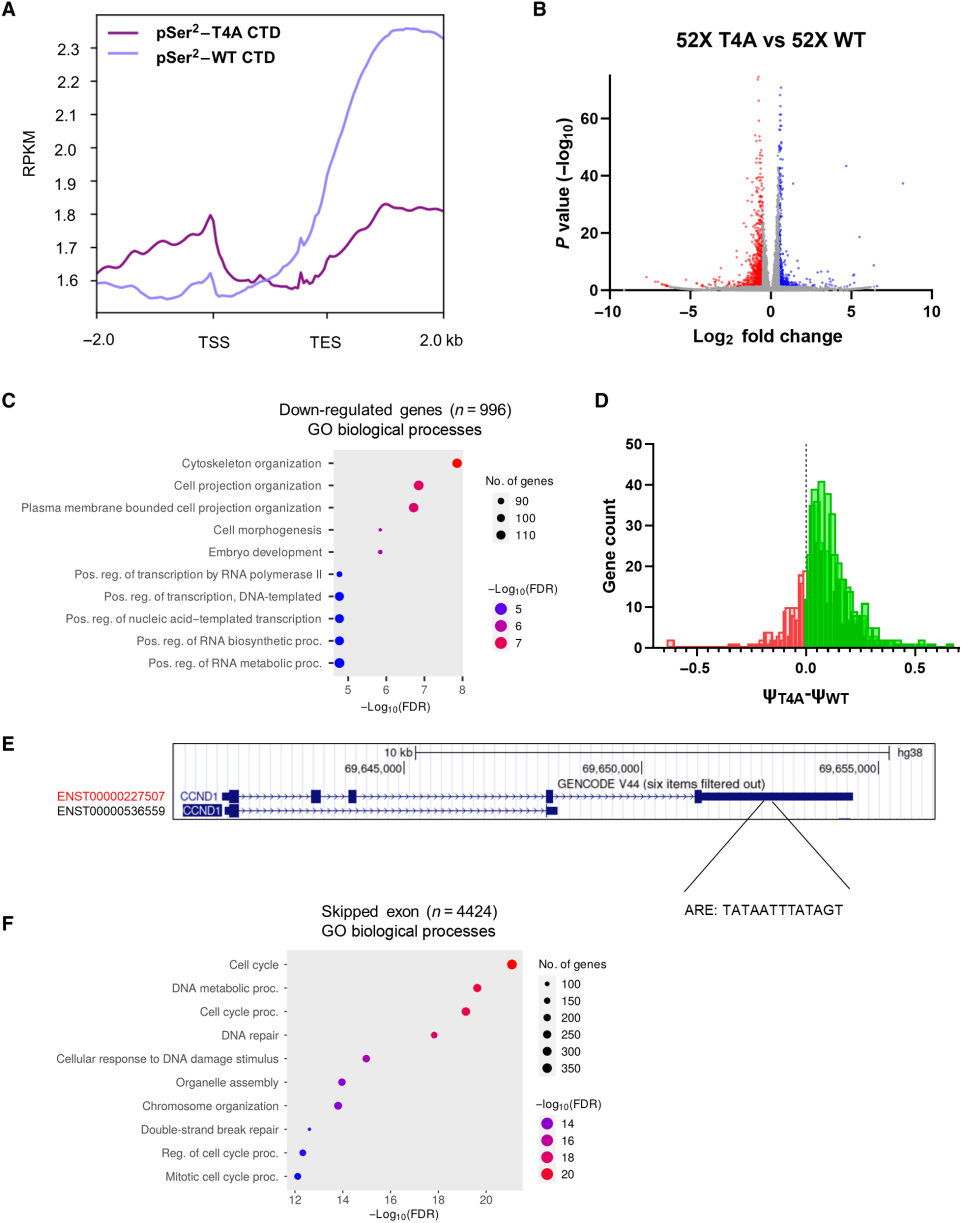
Function of Thr^4^ phosphorylation in 3′ processing. (**A**) Normalized pSer^2^ ChIP signal along human genes when T4A or WT Pol II CTD is expressed. (**B**) Volcano plot showing the log fold change (thresholds of 1.5 and −1.5) of gene expression changes due to 52X T4A CTD expression with an adjusted *P* value threshold of 0.05. (**C**) GO analysis of the above significant negatively regulated genes upon 52X T4A-CTD expression. (**D**) Histogram of comparisons between significant ψ values in cells expressing 52X T4A or 52X WT RNA Pol II CTD (FDR < 0.05). Positive ψ values are shown in green, and negative ψ values are shown in orange. (**E**) Example of the gene (*CCND1*) found to prefer distal poly(A) site usage when transcribed. Difference between proximal and distal poly(A) sites estimated by LABRAT is 6234 bp. (**F**) GO analysis of biological processes for transcripts with significant exon inclusion and exclusion events.

With T4 and S2 exhibiting overlapping interactomes, we considered the possibility that Thr^4^ and Ser^2^ phosphorylation cross-talk. Because pThr^4^ seems to occur at an earlier stage of transcription (Figs. 2B and 3A), we inquired if the level of Thr^4^ phosphorylation affected the phosphorylation of Ser^2^. To address this issue, we transiently transfected plasmids containing a WT RPB1 or a variant with all Thr^4^ replaced by Ala. To eliminate the interference of endogenous RPB1, we introduced a mutation into each plasmid, rendering the introduced protein resistant to α-amanitin. We established a concentration of α-amanitin that eliminated endogenous RPB1 and monitored the expression of either EYFP-RPB1 mutants using Western blot (WB) to ensure equal levels of RPB1 (T4A versus WT CTD) between conditions (fig. S6, A to C). With the exogenous WT RPB1 and T4A expressed at similar levels, we treated the cell with α-amanitin to eliminate the endogenous Pol II. After 48 hours of treatment, we collected the cells and analyzed the genomic location of the pSer^2^ in RPB1. We noticed that the pSer^2^ distribution varied greatly upon T4A mutation with a greatly reduced level of phosphorylation (Fig. 6A). Unlike the substantial accumulation of Ser^2^ phosphorylation close to the TES in normal RPB1, the loss of Thr^4^ phosphorylation in the RPB1 T4A variant suppresses Ser^2^ phosphorylation, especially at the TES region. Thus, the phosphorylation of Thr^4^ affects the pattern of Ser^2^ phosphorylation. The cross-talk between the two marks deserves further investigation and demands reevaluation of previous observation attributed to Ser^2^ phosphorylation to consider the possibility that it is the secondary effect from Thr^4^ phosphorylation.

### Effects of Thr^4^ phosphorylation on transcription

Thr^4^ phosphorylation has been implicated in cell cycle regulation (*12*) where Thr^4^ mutations display serious mitotic defects such as multipolar spindles and polyploid cells (*12*). Furthermore, Thr^4^ modification might also play a role in transcription-coupled DNA repair (*26*). To understand how Thr^4^ phosphorylation affects transcription, we characterized transcriptional alterations in mammalian cells expressing an RPB1 T4A variant after we removed endogenous Pol II through α-amanitin elimination for 48 hours. The reproducibility of biological replicates was high as the 52X T4A and 52X WT duplicates clustered together with a strong correlation coefficient (fig. S6D). In terms of the differentially expressed genes (DEGs), there were 996 down-regulated genes (|fold change| > 1.5 and adj. *P* value < 0.05) and 257 up-regulated genes in 52X T4A cells (Fig. 6B and table S3). Pathway analysis reveals that down-regulated genes include an abundance of those playing a role in cytoskeleton organization (Fig. 6C). This is consistent with previous results showing that mitotic Pol II at centrosomes exclusively retains pThr^4^ marks and mutations at this position alter mitotic division (*12*).

Our mechanistic study indicates that the CID-containing proteins identified to be recruited to Pol II through Thr^4^/Ser^2^ phosphorylation and these proteins mostly play a role in a proper 3′ untranslated region (3′UTR) in the transcriptome (*30*, *38*). The sequence content of the 3′UTR region can vary greatly depending on if a more proximal or distal poly(A) site is used to terminate the transcript (*39*). The 3′UTR maintains the stability of the transcript by housing regulatory regions that determine mRNA localization, binding regions for RNA binding proteins, miRNA recognition sites, and AU-rich elements (AREs) that promote mRNA decay in a controlled manner (*40*, *41*). Misregulation of poly(A) site usage can lead to removal or addition of binding sites, thereby altering the metabolism of the mRNA product (*42*). To ascertain the global impact of pThr^4^ on polyadenylated transcripts, we compared APA site usage of cells containing T4A or WT Pol II α-amanitin mutants by the LABRAT method (*43*). APA analysis showed a significant increase in distal poly(A) usage, denoted by a positive Δ-Ψ value, in 573 genes when 52X T4A RPB1 is expressed compared to 52X WT, whereas only 140 genes showed the opposite trend (Fig. 6D, fig. S6E, and table S4). Gene ontology (GO) analysis for the 573 genes with increased 3′UTR lengths revealed processes that maintain intracellular transport and mitotic cell cycle progression among the top 20 enriched pathways (table S4).

To further dissect the effects of distal poly(A) site usage in the T4A Pol II mutant on its phenotype, we used the ARED-Plus database to predict genes with AREs in their 3′UTRs (*44*). The analysis showed that one-third of the genes (181/573) favoring distal poly(A) sites contained predicted AREs in 3′UTR. In addition, 14% of this overlap was simultaneously found to be down-regulated by DEG analysis (tables S1 and S3), which supports a notion about the decreased stability of ARE-containing transcripts. We followed up on one of the most significant and large GO categories among distal poly(A) genes, “Cell cycle process” [66 genes, fold change = 1.9, false discovery rate (FDR) = 0.00014] (table S4). Using this gene subset as a query against database (*44*), we found that 19 of 66 genes were predicted to contain AREs in their 3′UTR (table S4). Among these genes, there were important pro-proliferative mediators such as cyclins CCND1 and CCND3, cyclin-dependent kinase CDK2, mitotic checkpoint protein BUB3, and NEK7 kinase, a regulator of cell division. In addition, two genes in this group—microtubule stabilizer *CLASP2* and huntingtin *HTT*—were also down-regulated based on DEG analysis. By querying the LABRAT output against the transcript annotation from the UCSC Genome Browser (hg38), we confirmed the presence of ARE sequences in the longest transcripts of these genes with distal poly(A) sites and the lack of thereof in the transcripts favoring proximal poly(A) sites (*CCND1* as an example is shown in Fig. 6E). Therefore, distal poly(A) site usage could add AU-rich sequences in mRNA transcripts of genes important for cell cycle progression that may eventually lead to AU-mediated decay and proliferation arrest. This is consistent with the reported phenotype that the T4A mutant affects cell cycle regulation (*12*). Distal poly(A) site usage is reminiscent of termination defects in budding yeast where Pol II is detected transcribing far downstream at snoRNA termination sites when T4 is mutated (*13*). Likewise, a T4V substitution also displays delayed dissociation of Pol II at termination sites with a downstream shift in positions of poly(A) sites in budding yeast (*14*).

Although the CTD itself does not directly interact with the core components of spliceosome, the presence of CTD is believed to promote spliceosome assembly (*45*). To investigate the effects of pThr^4^ specific roles on the selection of splicing site, we conducted deep sequencing of RNA transcription to examine five major alternative splicing events (ASEs) by the rMATS tool (*46*). The alternative splicing analysis revealed 6359 unique isoform changes across five different event types (fig. S6F and table S5) with a significance threshold of FDR < 0.05 and inclusion level difference (ILD) ≥ 10% between experimental groups. Although variations are identified in each type of ASE, there was no obvious trend to favor inclusion or exclusion of certain exons or introns. However, GO enrichment analysis of transcripts with ASEs highlighted biological processes such as “mitotic cell cycle” and “chromosome segregation” to be significantly overrepresented (Fig. 6F). Dysregulation of the mitotic cell cycle could emanate from alternative splicing changes in transcripts involved in its maintenance of which phosphorylation at Thr^4^ controls.

Overall, our data suggest an important functional role of Thr^4^ phosphorylation in transcripts’ 3′-end processing, specifically in the preferential choice of distal poly(A) sites that potentially leads to addition of binding sites, such as AREs, and decreased stability of the transcripts.

## DISCUSSION

The role of Thr^4^ within the highly conserved sequence of the CTD heptad in RNA Pol II has long confounded researchers. Its significance becomes apparent as its replacement induces severe defects in elongation, termination, and processing, ultimately culminating in mammalian cell death (*6*, *7*). However, despite its crucial role in early transcriptional events, Thr^4^ phosphorylation marks remain undetected until the conclusion of transcriptional events, typically occurring 500 to 2000 bp downstream of polyadenylation sites (*16*, *47*). Scientists have pondered the possibility that this contradiction may stem from the strong masking effect of neighboring Ser^5^ phosphorylation on the pThr^4^ antibodies at the onset of the transcription cycle (*47*). Although pThr^4^ antibodies exhibit high specificity, they are susceptible to interference from Ser^5^ phosphorylation (*16*), a prevalent event during transcription initiation.

Our use of phosphatase complex, Ssu72/symplekin, to eliminate masking Ser^5^ phosphorylation revealed a pronounced peak near the TSS in nearly all expressed protein-coding genes across all six datasets. While a previous study detected a minor peak at the TSS in the top 5% of genes, our study identified the pThr^4^ peak in nearly 10,000 protein-coding genes (*16*). The emergence of the previously unidentified pThr^4^ peak near the TSS elucidates the involvement of pThr^4^ in elongation, consistent with genetic and mutagenesis studies of T4A cells. Previous investigations have demonstrated that T4A substitution within the CTD prompts RNA Pol II to stall at the TSS (*16*). Our ChIP analysis supports this observation, indicating that significant Thr^4^ phosphorylation initiates precisely when productive elongation should commence. Consequently, the inability to phosphorylate Thr^4^, as observed in T4A mutants, hinders Pol II from entering productive elongation, underscoring the critical role of Thr^4^ phosphorylation in transcriptional dynamics. The transcriptomic analysis also unveils the molecular mechanism behind T4A lethality and cell cycle regulation (*12*). Upon T4A mutation, the genes involved in mitotic cell cycle regulations are affected in a multilayer manner. The foremost category of down-regulated genes is involved in cytoskeleton organization (Fig. 6C), the cell cycle genes altered to a distal poly(A) site (Fig. 6D), and alternations in splicing events lead to changes of cell cycle genes (Fig. 6F).

Our study uncovered a distinctive property of pThr^4^ not observed in other Pol II PTMs: Its genomic profile varies among gene types, suggesting a gene-specific CTD code. Analysis of ChIP-seq results reveals a consistent distribution profile of pThr^4^ among protein-coding genes, whereas noncoding genes such as snoRNAs exhibit a distinct profile characterized by a broad peak around the TSS with no significant association at the TES (see Fig. 2E). These distinct profiles imply that the role of T4 phosphorylation may differ between these gene groups, highlighting a gene-specific CTD coding system. Given the association of pThr^4^ alteration with environmental cues (*17*, *18*), it may serve as a mark specifically reserved for stress response via snoRNA regulation. The T4A variant in yeast caused significant changes in snoRNA expression (*13*, *26*). Investigating if the distribution of T4 phosphorylation varies across gene categories thus holds substantial interest and could illuminate evolutionary adaptations in transcriptional regulation.

The similarity between the pThr^4^ phenotype and function lies in its resemblance to pSer^2^. Their ChIP profiles exhibit notable similarities, and the proteins recruited by this mark to RNA Pol II are nearly identical as found in proteomic studies from our and other labs (*14*, *27*). Our ChIP mechanistic study reveals cross-talk between the two, wherein pThr^4^ influences the level of Ser^2^ phosphorylation. The crucial occurrence of pThr^4^ at the outset of transcription raises the intriguing possibility that many factors previously attributed to Ser^2^ phosphorylation function may deserve to be credited to pThr^4^ instead. For instance, a family of proteins containing a motif (CID) for CTD recognition, traditionally known to bind pSer^2^, demonstrates similar, sometimes even tighter, binding to pThr^4^ in vitro, with a ChIP recruitment profile more consistent with pThr^4^. Notably, these factors predominantly play roles in 3′-end processing. Given pThr^4^’s earlier appearance in the transcription cycle and its potent binding to regulatory proteins, it is prudent to reconsider the previous characterization of Ser^2^ as one of the two major PTM marks in eukaryotic transcription, potentially overlooking the collaborative relationship of Ser^2^ and Thr^4^. Our ChIP-seq studies of T4A Pol II provide evidence that loss of phosphorylation at this site lowers levels of Ser^2^ phosphorylation. Considering that both marks are phosphorylated by the positive transcription elongation factor b (P-TEFb), which is recruited to Pol II by a mediator and BRD4 close to the TSS, the new ChIP profile of pThr^4^ necessitates a reassessment of the functions traditionally ascribed to pSer^2^, suggesting that Thr^4^ phosphorylation may substantially contribute to processes originally solely attributed to pSer^2^.

## MATERIALS AND METHODS

### Cell culture

HEK293 cells were purchased from the American Type Culture Collection (Manassas, VA, United States). Cells were routinely cultured in Dulbecco’s modified Eagle’s media (Sigma-Aldrich, St. Louis, MO, United States, product no. D6429), supplemented with 10% Opti-Gold fetal bovine serum (GenDEPOT, Katy, TX, United States) at 37°C in humidified atmosphere with 5% CO_2_. HyClone penicillin and streptomycin mix (Cytiva, Marlborough, MA, United States) was added to the media to reach a final concentration of 1%.

### Cloning

The 26X T4E-CTD and 52X T4A substrate was ordered as a synthetic gene and cloned into a pET28a (Novogene, Sacramento, CA, United States) derivative vector encoding a 6xHis tag followed by a glutathione *S*-transferase (GST) tag and a 3C protease site or an N-terminal HA-tagged mammalian expression vector. The RPRD1B-CID (encoding residues 2 to 133), RPRD1A-CID (residues 2 to 133), RPRD2-CID (residues 19 to 149), and SCAF4-CID (residues 1 to 139) and Dyrk1a kinase domain (residues 127 to 485) were ordered as synthetic genes. The full-length RPRD1B cDNA (clone: HG14027-G) encoding residues 1 to 326 was cloned into a mammalian expression vector containing a cytomegalovirus promoter and an N-terminal HA tag. The 52X WT CTD harboring α-amanitin resistant and an EYFP tag was from Addgene (plasmid no. 75284). Drosophila Ssu72 (1 to 195) and symplekin (residues 19 to 351) were cloned into a pET28b vector encoding a 6xHis tag and Small Ubiquitin-like Modifier (SUMO) tag.

### Protein expression and purification

For protein expression, BL21 (DE3) cells expressing RPRD1B-CID, RPRD1A-CID, RPRD2-CID, SCAF4-CID, Dyrk1a, or GST-CTD substrates were grown in 1-liter cultures at 37°C in Luria-Bertani (LB) broth (Thermo Fisher Scientific, Waltham, MA, United States) containing kanamycin (50 μg/ml). Once the cultures reached an OD 600 (optical density at 600 nm) value of 0.6 to 0.8, the protein expression was induced with 0.25 mM isopropyl-β-d-thiogalactopyranoside, and the cultures were grown for an additional 16 hours at 18°C. The cells were pelleted and resuspended in a lysis buffer [50 mM tris-HCl (pH 8.0), 500 mM NaCl, 15 mM imidazole, 10% glycerol, 0.1% Triton X-100, and 10 mM 2-mercaptoethanol (BME)] and sonicated at 90 A for 2.5 min of 1-s on/5-s off cycles on ice. The lysate was cleared by centrifugation at 15,000 rpm for 45 min at 4°C. The supernatant was loaded over 3 ml of Ni-NTA beads (Qiagen, Germany) equilibrated in a lysis buffer then washed with a wash buffer containing 50 mM tris-HCl (pH 8.0), 500 mM NaCl, 30 mM imidazole, and 10 mM BME. The recombinant protein was eluted with a buffer containing 50 mM tris-HCl (pH 8.0), 500 mM NaCl, 300 mM imidazole, and 10 mM BME. Protein fractions were pooled and dialyzed overnight at 4°C in a 10.0-kDa dialysis membrane (Thermo Fisher Scientific) against a dialysis buffer [50 mM tris-HCl (pH 7.5), 100 mM NaCl, and 10 mM BME]. The protein was polished using gel filtration chromatography and loaded onto a Superdex 75 or 200 size exclusion column (GE) in a gel filtration buffer. For Ssu72 and symplekin, the individual proteins were concentrated, combined, and dialyzed overnight followed by size exclusion chromatography of the complex. Peak fractions were analyzed by SDS.

### Dot blot

Serial dilutions of pThr^4^ CTD peptide or Dyrk1a/Abl1 kinase-treated pThr^4^ CTD peptide were spotted on an activated nitrocellulose membrane. The membrane was allowed to dry and blocked by soaking in 5% bovine serum albumin (BSA)/Tris-buffered saline with 0.1% Tween 20 detergent (TBS-T) for 30 min at room temperature. The membrane was then incubated with primary antibody (1:1000) in BSA/TBS-T for 30 min at room temperature. The membrane was washed three times with TBS-T for 5 min each. Then, the membrane was incubated with secondary antibody (1:10,000) for 30 min at room temperature. The membrane was washed three times and visualized on a LI-COR Odyssey CLx image reader.

### Western blot

Cells were lysed in a radioimmunoprecipitation assay lysis buffer [50 mM tris-HCl (pH 8.0), 150 mM NaCl, NP-40, 0.5% sodium deoxycholate, and 0.1% SDS] and 1× protease inhibitor cocktail (Roche, Indianapolis, IN, United States). Protein concentrations were quantified with the Bradford protein assay. Briefly, 25 μg of protein extracts was loaded and separated by SDS–polyacrylamide gel electrophoresis gels. Blotting was performed with standard protocols using a PVDF membrane (Bio-Rad, Hercules, CA, United States). Membranes were blocked for 1 hour in a blocking buffer [5% BSA in phosphate-buffered saline with Tween detergent (PBST)] and probed with primary antibodies at 1:1000 dilution at 4°C overnight. After three washes with PBST, the membranes were incubated with diluted goat anti-rabbit or anti-rat secondary IRDye 680RD antibody at 1:10,000 (LI-COR, Lincoln, NE, United States) for 1 hour at room temperature. After washing, membranes were visualized on the LI-COR Odyssey CLx image reader. For WB or dot blot analysis, phospho-specific antibodies, pThr^4^ (catalog no. 61361; 1:100 to 1:800 dilution for WB and IF) and pSer^2^ (Sigma-Aldrich, stock keeping unit (SKU): MABE953; 1:1000 dilution for WB and IF), green fluorescent protein (GFP) antibody (catalog no. 50430-2-AP; 1:1000 dilution), and HA antibody (catalog no. C29F4; 1:1000 dilution for WB and 1:800 for IF) were used. The RPRD1B antibody is from Cell Signaling Technology (catalog no. 74693; 1:1000 dilution for WB).

### Coimmunoprecipitation

Cellular extracts were prepared by incubating cells with a lysis buffer [50 mM tris-HCl (pH 8.0), 150 mM NaCl, 0.5% NP-40, 1 mM phenylmethylsulfonyl fluoride, and 1× protease inhibitor) for 30 min on ice. The supernatant was collected by centrifugation at 12,000*g* for 20 min at 4°C. For immunoprecipitation, Dynabeads Protein A (20 μl, Invitrogen) was incubated with 2 μg of antibody overnight at 4°C with rotation. Subsequently, 250 μg of protein was incubated with the antibody-bound beads for an additional 2 hours and washed three times with a lysis buffer. The precipitated proteins were eluted from the beads with a 2× SDS loading buffer and boiled for 5 min, followed by WB analyses. Three independent replicates of each IP experiment were performed.

### Immunofluorescence

In brief, HEK293 cells were transfected with HA-RPRD1B and 52X T4A Pol II or 52X WT Pol II using polyethylenimine (PEI) (1:7 plasmid-to-reagent ratio) to overexpress the protein of interest. Cells were washed with phosphate-buffered saline (PBS) and fixed in 1% formaldehyde for 15 min at room temperature. Cells were permeabilized with 0.2% Triton X-100 to allow antibody labeling. Subsequently, the samples were blocked with 2% BSA for 30 min and incubated with primary antibody for 1 hour at room temperature. After washing with PBS, the cells were stained with secondary antibody [goat anti-rabbit immunoglobulin G (IgG) (H+L) cross-adsorbed secondary antibody Alexa Fluor 488 or goat anti-rat IgG (H+L) cross-adsorbed secondary antibody Alexa Fluor 568, Thermo Fisher Scientific] for 1 hour at room temperature. Cells were counterstained with 4′,6-diamidino-2-phenylindole (DAPI) for nuclear visualization, and coverslips were mounted with antifade fluorescent mounting media (Abcam, catalog no. ab104135). Standard fluorescence images were captured using a confocal microscope (Zeiss LSM 710). Confocal images were acquired with the Plan-Apo 63x oil immersion lens and analyzed using the Zen/ImageJ program.

### Crystallization

Initial crystallization conditions for RPRD1B-CID with pThr^4^ CTD peptide were identified using sparse-matrix screening using a Phoenix crystallization robotic system (Art Robbins Instruments). The identified hits for crystallization were optimized systematically using the sitting drop vapor diffusion technique. The complex structure was crystallized in 20 to 32% PEG-3350 (polyethylene glycol, molecular weight 3350), 0.1 M lithium sulfate, and a 1:3 molar ratio of protein to peptide. In all crystallization setups, a protein solution (~20 mg/ml) was mixed with an equal volume of the reservoir solution and equilibrated against 500 μl of the reservoir at room temperature. All crystals were cryoprotected with mother liquor supplemented with 30% glycerol and flash-frozen in liquid nitrogen.

### Data collection, processing, structure determination, and refinement

X-ray diffraction data for the RPRD1B-pThr^4^ structure were collected at the Advanced Photon Source beamline 23-ID-D (Argonne National Laboratories). The datasets were indexed, integrated, and scaled using HKL-2001 (*48*). The structures were determined by molecular replacement (MR) using Phase-MR2 from the PHENIX Suite of program (*49*). One monomer of the RPRD1A-CID (PDB: 4JXT) was used as a search model for the initial phases. Structure refinement was performed using phenix.refine along with iterative model building in COOT (*50*). TLS parameters were included in the refinement of all structures. The final structures were evaluated after refinement using MolProbity (*51*). The refinement statistics for the structures are summarized in table S5. All figures were prepared with PyMol (The PyMOL Molecular Graphics System, version 1.8, Schrödinger LLC).

### Phosphorylation sample preparation

Kinase reactions were performed in a buffer containing 2 mM adenosine 5′-triphosphate, 50 mM Tris (pH 8.0), and 10 mM MgCl_2_ and supplemented with the CTD substrate (1 mg/ml) for 15 hours. Reactions were initiated by adding 0.6 μM Dyrk1a. The reaction time was optimized so that no further phosphorylation occurred on the substrate. Reactions were quenched with the addition of 10 mM EDTA.

### Label-free proteomics sample preparation and CTD affinity purification

Dyrk1a (0.6 μM) was used to phosphorylate the 26x yeast GST-CTD substrate (1 mg/ml) in a 100-μl reaction for 15 hours. Likewise, a 26x yeast GST-T4E CTD substrate was incubated in a similar manner without any kinase treatment. Glutathione Agarose beads were washed thrice in buffer C [20 mM Tris (pH 8.0), 150 mM NaCl, and 10 mM BME], and the treated GST-CTD samples were added to the beads and incubated overnight. A total of 200 million HEK293 cells were grown, collected, and the cell pellet was resuspended in buffer A [10 mM Hepes (pH 7.4), 100 mM NaCl, 300 mM sucrose, 3 mM MgCl_2_, 0.5% Triton X-100, 1:100 protein, and phosphatase inhibitor]. Cells were then vortexed, incubated on ice for 15 min, and centrifuged at 15,000*g* for 10 min at 4°C. The supernatant is discarded, and the cell pellet was resuspended in buffer B [10 mM Tris (pH 8.0), 150 mM NaCl, and 1:100 protease and phosphatase inhibitors (PPI)] supplemented with 1:1000 benzonase. This mixture was incubated at room temperature for 1 hour and centrifuged at 15,000*g* for 10 min. The supernatant was collected as the nuclear fraction. After overnight incubation, the GST-CTD bound beads were washed twice with buffer C and once with buffer B. The nuclear fraction was added to the substrate-bound beads and incubated at 4°C overnight. Then, the beads were centrifuged at 4000*g* for 2 min at 4°C. The beads were washed twice with low salt buffer [20 mM Tris (pH 8.0), 150 mM NaCl, 10% glycerol, 0.1% Triton X-100, and 1:100 PPI] for 5 min per wash and thrice with high salt buffer [20 mM Tris (pH 8.0), 500 mM NaCl, 10% glycerol, 0.1% Triton X-100, and 1:100 PPI]. To the beads, 100 μl of elution buffer was added and spun at 4°C for 2 hours. Then, the beads were centrifuged at 4000*g* for 2 min at 4°C, and the supernatant was collected for the pulldown.

Pulldown samples were exchanged into 5 mM tris-HCl using 3-kDa Amicon filters. Samples were then denatured in 2,2,2-trifluoroethanol and 5 mM tris(2-carboxyethyl)phosphine at 55°C for 45 min. Proteins were alkylated in the dark with 5.5 mM iodoacetamide, and the remaining iodoacetamide was quenched with 100 mM dithiothreitol. MS-grade trypsin was then added to the solution at an enzyme:protein ratio of 1:50, and the digestion reaction was incubated at 37°C for 4 hours. Trypsin was quenched by adding 10% formic acid, and the volume was reduced to 500 μl in a vacuum centrifuge. Samples were then filtered using a 10-kDa Amicon filter and desalted using Pierce C18 tips (Thermo Fisher Scientific). The samples were resuspended in 95% water, 5% acetonitrile, and 0.1% formic acid prior to MS.

### Proteomics MS and protein identification

Peptides were separated on an Acclaim PepMap100 C-18 column (75 μM × 25 cm; Thermo Fisher Scientific) using a 5 to 50% acetonitrile + 0.1% formic acid gradient for 120 min and analyzed online by nanoelectrospray ionization tandem MS on a Thermo Fisher Scientific Fusion Tribrid Orbitrap mass spectrometer, using a data-dependent acquisition strategy and analyzing two biological replicates per sample. Full precursor ion scans (MS1) were collected at a high resolution (120,000). MS2 scans were acquired in the ion trap in rapid scan mode using the Top Speed acquisition method and fragmenting by collision-induced dissociation. Dynamic exclusion was activated with a 60-s exclusion time for ions selected more than once.

Proteins were identified with Proteome Discoverer 2.3 (Thermo Fisher Scientific), searching against the UniProt human reference proteome. Methionine oxidation [+15.995 Da], N-terminal acetylation [+42.011 Da], N-terminal methionine loss [−131.04 Da], and N-terminal methionine loss with the addition of acetylation [−89.03 Da] were all included as variable modifications. Peptides and proteins were identified using a 1% FDR.

To score changes in protein abundance, a *z* score was estimated between the unmodified control and the kinase-treated sample for each protein as in (*52*). To generate volcano plots, datasets from both replicates were log_2_ transformed, missing values were imputed using fancyimpute version 0.7.0, and data were quantile normalized. Enriched proteins were defined using a *P* value of 0.05. *P* values in volcano plot analyses were calculated using a two-tailed, two-sample *t* test.

### Fluorescence polarization

CTD peptides with double repeats were labeled with fluorescein isothiocyanate (FITC) and purchased from Biomatik. Protein and peptide concentrations were determined according to their absorbance at 280 nm. Fluorescence polarization values were collected on a Tecan F200 plate reader in a buffer [50 mM Tris (pH 8.0), 300 mM NaCl, and 10 mM BME] at room temperature. Samples were excited with vertically polarized light at 485 nm and at an emission wavelength of 535 nm. RPRD1B-CID, RPRD1A-CID, RPRD2-CID, and SCAF4-CID protein was titrated into a reaction mixture containing a buffer supplemented with 10 nM FITC-peptide. Measurements were taken in triplicate, and the experimental binding isotherms were analyzed in GraphPad Prism v9 using a total binding mode to obtain *K*_d_ values.

### Reverse transcription qPCR

Total RNA was harvested from HEK293 or HEK293T cells using the DirectZol RNA Miniprep kit (Zymo Research, Irvine, CA, United States, product no. R2050). cDNA was generated using the AzuraQuant cDNA synthesis kit (Azura Genomics) following the manufacturer’s instructions. qPCR was done using the AzuraQuant Green Fast qPCR Mix Lo-Rox (Azura Genomics) in a ViiA-7 Real Time PCR system (Applied Biosystems). All qPCR experiments were conducted in biological triplicate; error bars represent means ± SEM. Relative gene expression was assessed using the ∆∆Ct method normalized to actin β (ACTB) expression. Student’s *t* test was used to compare groups. All primers used in this study can be found in table S7.

### Differential scanning fluorometry

Purified recombinant RPRD1B-CID at a final concentration of 5 μM was incubated with 10X SYPRO Orange (Molecular Probes) in a 96-well low-profile PCR plate (ABgene, Thermo Fisher Scientific), and fluorescence was captured in a LightCycler 480 (Roche). Protein melting curves were carried out with a temperature acquisition mode using a total of 10 acquisitions per 1°C in each cycle from 20° to 95°C. The melting temperature was derived using the Boltzmann equation.

### RNA isolation, library preparation, and RNA sequencing

Total RNA was isolated from HEK293T cells (at least ~10^6^ cells per sample) using the DirectZol RNA Miniprep kit (Zymo Research). RNA integrity was assessed by Novogene Co. using the RNA Nano 6000 assay kit of the Bioanalyzer 2100 system (Agilent Technologies, CA, United States). Libraries were prepared at Novogene Co. according to the manufacturer’s instructions for the NEBNext Ultra RNA Library Kit for Illumina. The resulting libraries tagged with unique dual indices were checked for size and quality using the Agilent Bioanalyzer 2100. Libraries were loaded for sequencing on the NovaSeq 6000 (Illumina, San Diego, CA, United States) instrument (paired-end 2X150).

### Analyses of RNA-seq data and APA

Quality of raw reads was assessed using FastQC read quality reports (https://usegalaxy.org) (*53*). Adapter Illumina sequences were trimmed off by Trimmomatic v.0.38 with default parameters (*54*). Next, reads were aligned to a human reference genome, GRCh38 version, using HISAT2 fast aligner v.2.2.1 with default parameters and –unstranded (*55*). The Gencode v38 gtf file was used as annotation gtf. Last, mapped fragments were quantified by featureCounts v.2.0.1 in Galaxy (*56*). Differential expression was analyzed using edgeR v.3.36.0; genes with FDR < 0.05 were considered as differentially expressed (*57*). RNA sequencing (RNA-seq) data were deposited in Gene Expression Omnibus (GEO) under the accession number GSE262702. Quantification of differential APA usage was performed using LABRAT (*43*). Tffasta was used to filter transcripts that have ill-defined 3′ ends, and the last two exons of each transcript were extracted. For --librarytype, RNA-seq was chosen. The 3′ ends were then quantified using Salmon. Calculatepsi was used to calculate the relative usage of these ends, compare across conditions, and Ψ values were calculated for each gene in each sample with an expression level cutoff of 5 Transcripts Per Million (TPM). Enrichment analysis of biological processes was performed with ShinyGO v.0.80 (*58*). ARE search in sequences of select subset of genes preferring distal poly(A) sites (Ψ52xT4A-Ψ52xWT > 0) was performed using the ARED-Plus database (*44*).

### Analyses of ASEs

rMATS turbo v.4.1.2 was used for detection of five major alternatively spliced events upon 52X T4A vs. 52X WT expression (with parameters, --libType set to unstranded, FDR < 0.05; ILD ≥ 10%) (*46*). As input files for rMATS, alignment .bam files from HISAT2 mapper and gencode v38 annotation gtf were used.

### ChIP and ChIP-seq

To generate 52X T4A and 52X WT CTD for ChIP studies, transient transfection of 12 μg of either plasmid was performed using PEI. Following transfection, α-amanitin (2.5 μg/ml) was added to cells for 48 hours. Briefly, for HA-tagged proteins, HEK293 cells were double cross-linked with 2 mM disuccinimidyl glutarate (DSG) for 15 min followed by secondary fixation with 1% formaldehyde for 10 min at room temperature. Single cross-linking was used for RPB1 ChIP using 1% formaldehyde for 10 min. Cross-linking was quenched with 0.125 M glycine for 5 min. Cells were successively lysed in lysis buffer LB1 [50 mM Hepes-KOH (pH 7.5), 140 mM NaCl, 1 mM EDTA, 10% glycerol, 0.5% NP-40, 0.25% Triton X-100, and 1× PPI), LB2 [10 mM tris-HCl (pH 8.0), 200 mM NaCl, 1 mM EDTA, 0.5 mM EGTA, and 1× PPI], and LB3 [10 mM tris-HCl (pH 8.0), 100 mM NaCl, 1 mM EDTA, 0.5 mM EGTA, 0.1% Na deoxycholate, 0.5% *N*-lauroylsarcosine, and 1× PI]. Chromatin was sonicated to an average size of ~200 to 500 bp using a UCD-200 Biorupter (30-s on and 30-s off for 30 min). A total of 5 μg of HA antibody (catalog no. C29F4), pThr^4^ antibody (Active Motif, catalog no. 61361), or pSer^2^ antibody (Sigma-Aldrich, SKU: MABE953) was premixed in a 50-μl volume of Dynabeads Protein A or Protein G (Invitrogen) and was added to each sonicated chromatin sample and incubated overnight at 4°C. For pThr^4^ samples, the Ssu72/symplekin complex (55 μM) was added to sonicated chromatin and incubated at 28°C for 30 min before immunoprecipitation. The chromatin-bound beads were washed two times with low salt buffer [0.1% Na deoxycholate, 1% Triton X-100, 1 mM EDTA, 50 mM Hepes (pH 7.5), and 150 mM NaCl], once with high salt wash buffer [0.1% Na deoxycholate, 1% Triton X-100, 1 mM EDTA, 50 mM Hepes (pH 7.5), and 500 mM NaCl], once with LiCl wash buffer [250 mM LiCl, 0.5% NP-40, 0.5% Na deoxycholate, 1 mM EDTA, and 10 mM tris-HCl (pH 8.0)], and twice in Tris-EDTA (TE) buffer. The chromatin was reverse cross-linked overnight at 65°C with shaking at 750 rpm. After DNA extraction using phenol-chloroform, the DNA was resuspended in 10 mM tris-HCl (pH 8.0). The purified DNA was subjected to qPCR to confirm target region enrichment before moving on to deep sequencing library preparation. For sequencing, the extracted DNA was used to construct the ChIP-seq library using the NEBNext Ultra II DNA Library Prep Kit followed by sequencing with an Illumina NovaSeq X Plus system. For pThr^4^ datasets, libraries were sequenced with an Illumina HiSeq 3000.

### Analysis of ChIP-seq data

After the initial assessment of read quality, pThr^4^ (untreated samples), pSer^2^, and RPRD1B (HA tag) ChIP-seq data were mapped onto a human reference genome, hg38, with a Bowtie2 v. 2.5.0 aligner for paired-end reads using default parameters (*59*). pThr^4^ (treated with Ssu72) single-end ChIP-seq reads were mapped onto a human reference genome, hg19, using BWA v.0.7.17 (*60*). Coverage tracks in .bigwig format were generated from filtered.bam files (mapq > 20) and visualized in the IGV v.2.4.16 software (*61*).

After alignment, MACS2 v.2.2.7.1 in Galaxy (parameters: --broad; --broad-cutoff of *q* < 0.1 for pThr^4^) was used to call peaks for immunoprecipitation samples against an input (*62*). Bioconductor R package “chipseeker” v.1.18.0 was used for deriving the consensus pThr^4^-Ssu72 peakset and peak annotation using gencode hg19 gtf as a reference (*63*). For peak annotation, promoters were defined as (−1000 bp, +1000 bp from TSS) regions. The Gencode v38 gtf file was used as the annotation gtf for pSer^2^ and RPRD1B data. TSS/TES profiling was done using plotProfile on matrices generated with 50-bp bins using the computeMatrix function from the deeptools v.2.2.3 (*64*). Calculation of TSS/TES ratios was performed using values derived with --outFileNameMatrix parameter of computeMatrix function and custom R script. The TSS and TES for ratio estimation were defined as values of the bin with maximal signal in the first (bins 1 to 60) and second (bins 61 to 120) halves of the profile, respectively. Reproducibility of ChIP-seq replicates was assessed by Pearson correlation analysis using the plotCorrelation function and/or binding affinity heatmaps in the DiffBind R package (*64*, *65*). ChIP-seq data were deposited in GEO under the accession number GSE262826.

Single-end ChIP-seq data from PMID: 22549466 (samples GSM920945-pThr4 ChIP, GSM920947-Pol II ChIP in consensus T48_control, GSM920949-Pol II ChIP in T4A mutant, input) were reanalyzed using the following steps. After the initial QC step, raw reads were trimmed using default parameters of -trim-galore (v.0.6.3). BWA v.0.7.17 was used to align reads onto a reference genome (hg19). After filtering out low-quality alignments (mapq < 20), the “MarkDuplicates” tool from Picard v.2.18.2 was applied to reduce duplication levels. TSS/TES profiling was done using matrices generated with 50-bp bins using the computeMatrix function from the deeptools.

### Statistical analyses

Statistical analyses were performed using RStudio v4.0.5 and GraphPad Prism v9. Two-tailed, independent sample *t* test was used for comparing the two groups (if not stated otherwise). *P* < 0.05 was considered as significant. Correlations were assessed using two-tailed Pearson *r* coefficients. Protein bands were quantified and compared using the ImageJ software.
