# Supplementary material for: Thr4 phosphorylation on RNA Pol II occurs at early transcription regulating 3′-end processing
Source: Sci Adv. 2024 Sep 6;10(36):eadq0350. doi: 10.1126/sciadv.adq0350 (PMC11378909; doi:10.1126/sciadv.adq0350)
Supplement: Supplementary file 1 — Figs. S1 to S6 Tables S6 and S7 Legends for tables S1 to S5 [file sciadv.adq0350_sm.pdf]

Science Advances
AAAS

Supplementary Materials for

Thr^4 phosphorylation on RNA Pol II occurs at early transcription regulating 3′-end processing

Rosamaria Y. Moreno *et al.*

Corresponding author: Y. Jessie Zhang, jzhang@cm.utexas.edu

Sci. Adv. **10**, eadq0350 (2024)
DOI: 10.1126/sciadv.adq0350

The PDF file includes:

Figs. S1 to S6
Tables S6 and S7
Legends for tables S1 to S5

Other Supplementary Material for this manuscript includes the following:

Tables S1 to S5

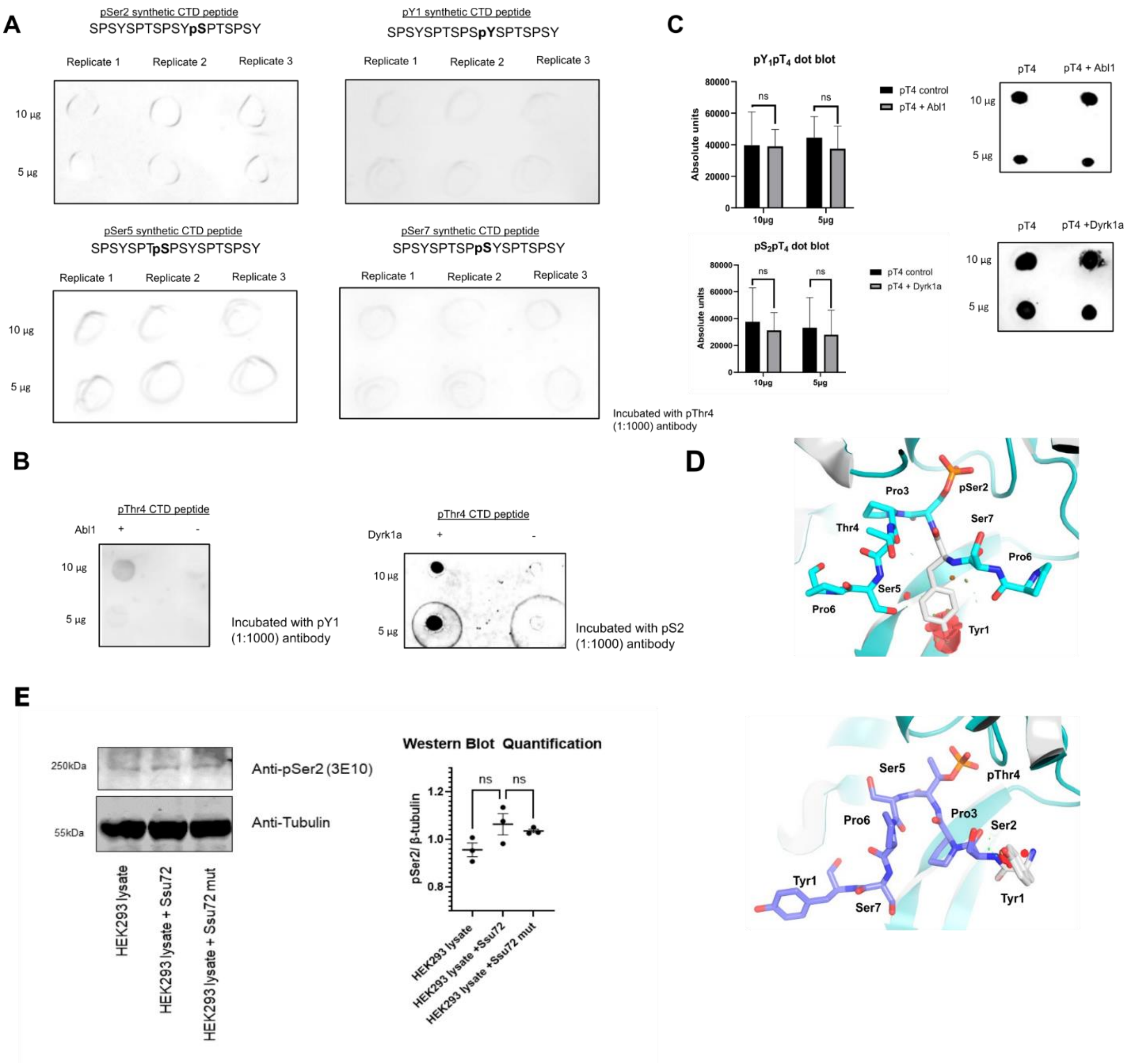

Fig. S1. Characterization of pThr4 antibody (clone 6D7) for ChIP analysis.

(**A**) Dot blot showing serial dilutions of a biotin-pSer2 , biotin-pY1, biotin-pSer5, and biotin-pSer7 CTD peptide incubated with pThr4 antibody for cross-reactivity analysis. **(B**) Dot blot showing singly pT4 peptide substrate treated with either Abl1 or Dyrk1a kinase and incubated with either pTyr1 or pSer2 antibody. (**C**) Dot blot showing serial dilutions Dyrk1a-treated pT4 CTD peptide vs no-kinase treated pT4 control peptide, and Abl1-treated pT4 vs no-kinase treated pT4 control peptide containing either 10µg or 5µg of peptide. Comparison analysis for all dot blots were performed using unpaired t-test. (**D**) Structural model of a pSer2 or pThr4 CTD peptide positioned in the active site of Ssu72 with steric clashes around Tyr1 shown. (**E**) Western blot showing pSer2 recognition in HEK293 cell lysate treated with Ssu72 or catalytically

inactive Ssu72. Comparison was performed using unpaired (Ssu72 WT vs untreated) or paired (Ssu72 WT vs Ssu72 Mut) t-tests. In all plots, mean with SEM are shown and quantification was from three independent biological replicates.

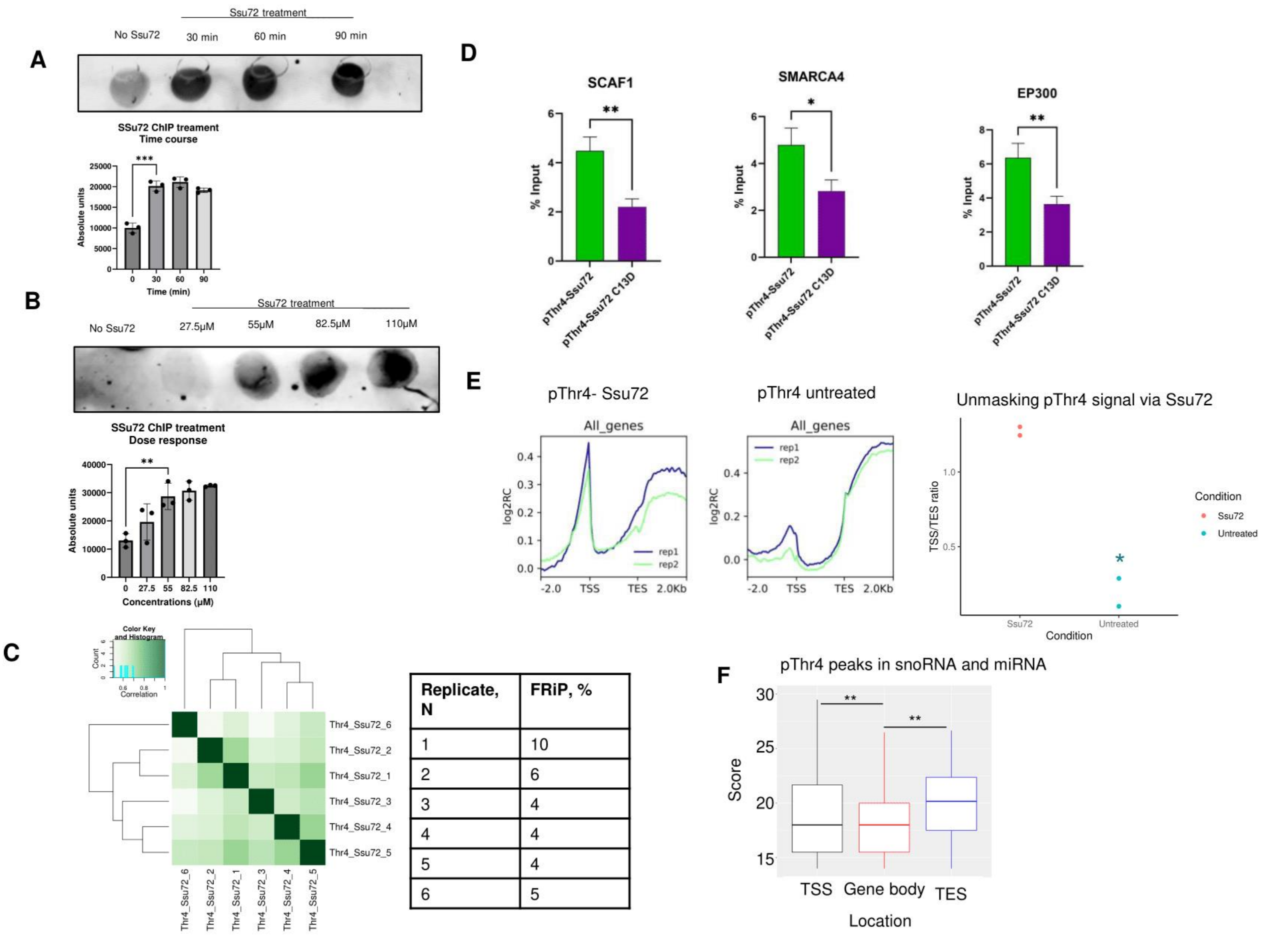

Replicate, N	FRiP, %
1	10
2	6
3	4
4	4
5	4
6	5

Fig. S2. ChIP-seq analysis of pThr4 Pol II samples treated with Ssu72.

(**A**) Dot blot showing a time course of chromatin isolated from HEK293T cells treated with Ssu72 for 30, 60 or 90 minutes and incubated with pThr4 antibody to detect a suitable reaction time for ChIP-seq (**B**) Dot blot showing a dose course of chromatin treated with Ssu72 at different concentrations and incubated with pThr4 antibody. Comparison was performed with unpaired t-test. (**C**) A binding affinity heatmap of 6 biological replicates of pThr4 ChIP-seq samples treated with Ssu72. FriP (Fraction of Reads in Peaks) scores are listed in the table. (**D**) ChIP-qPCR of gene regions enriched with pThr4 Pol II treated with Ssu72 WT compared to Ssu72 mutant. Shown are mean with SEM from 2-3 independent immunoprecipitations. Statistical comparison was performed using one-tailed t-test. **(E)** Comparison analysis of pThr4 ChIP without Ssu72 or with Ssu72. Unpaired one-tailed t-test was applied to the two biological replicates (reps1-2) to compare TSS/TES ratio of pThr4 ChIP signal. The y-axis corresponds to log2 of the ratio of the normalized counts between IP and input samples. TSS/TES values are

calculated using deeptools and coverage .bigwig files as the max signal surrounding TSS or TES over all genes. $^{**}p < 0.01$, $^{*}p < 0.05$. **(F)** Fold change distribution over TSS, gene body, and TES of sno/miRNA or LincRNA genes.

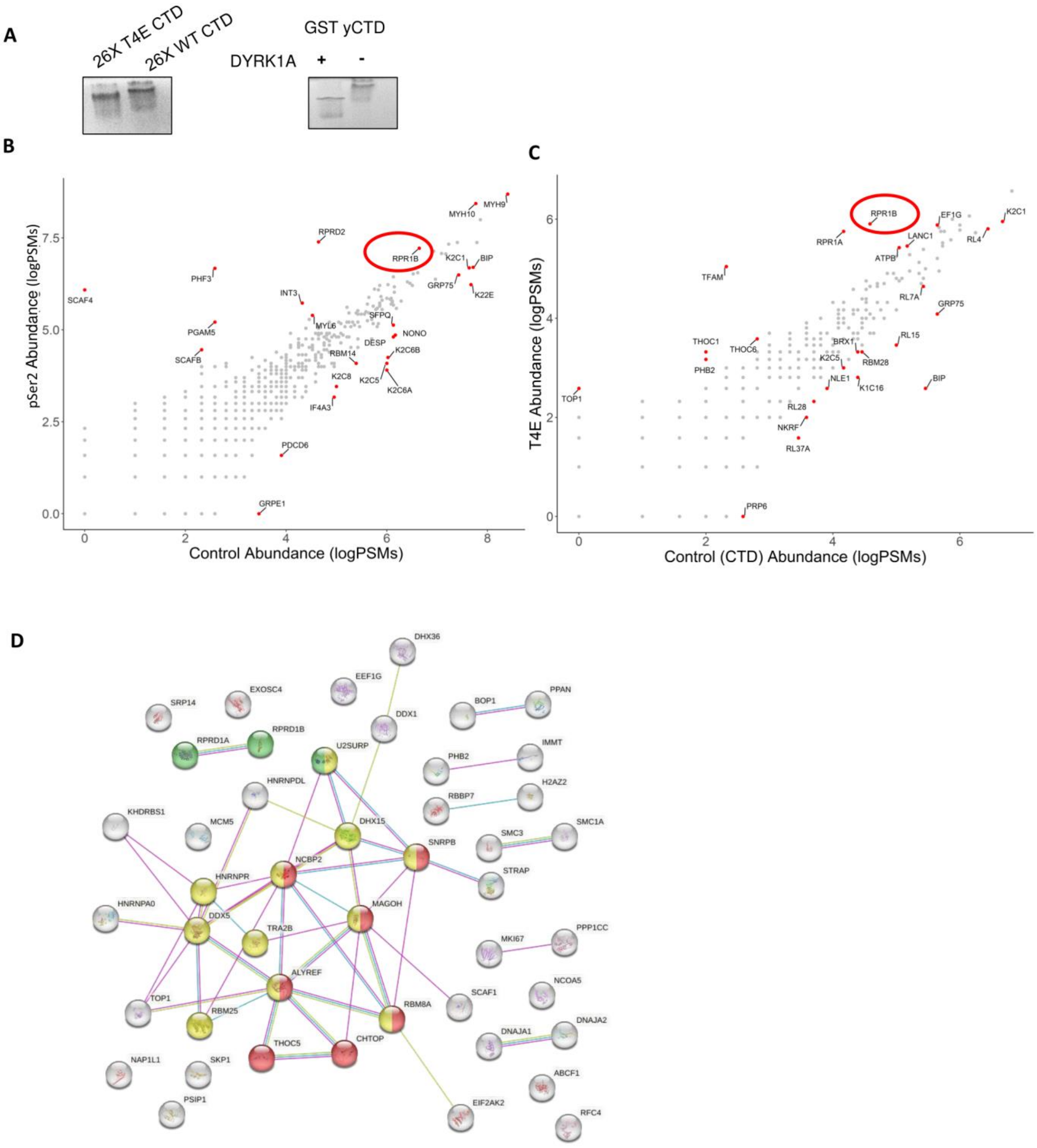

A
26X T4E CTD
26X WT CTD
GST yCTD
DYRK1A
+
-
B
pSer2 Abundance (logPSMs)
Control Abundance (logPSMs)
SCAF4
PHF3
PGAM5
SCAFB
RPRD2
INT3
MYL6
SFPQ
NONO
DESP
RBM14
K2C8
IF4A3
K2C5
K2C6B
K2C6A
PDCD6
GRPE1
RPR1B
MYH10
MYH9
K2C1
BIP
GRP75
K22E
C
T4E Abundance (logPSMs)
Control (CTD) Abundance (logPSMs)
TOP1
TFAM
THOC1
PHB2
THOC6
PRP6
RPR1B
RPR1A
EF1G
K2C1
LANC1
ATPB
RL4
RL7A
GRP75
RL15
BRX1
K2C5
RBM28
NLE1
K1C16
BIP
RL28
NKRF
RL37A
D
DHX36
EEF1G
SRP14
EXOSC4
BOP1
PPAN
DDX1
RPRD1A
RPRD1B
U2SURP
PHB2
IMMT
RBBP7
H2AZ2
HNRNPDL
KHDRBS1
MCM5
DHX15
SNRPB
SMC3
SMC1A
NCBP2
STRAP
HNRNPR
HNRNPA0
DDX5
TRA2B
MAGOH
MKI67
PPP1CC
ALYREF
TOP1
SCAF1
NCOA5
RBM25
RBM8A
DNAJA1
DNAJA2
THOC5
CHTOP
NAP1L1
SKP1
ABCF1
PSIP1
EIF2AK2
RFC4

Fig. S3. Identification of RPRD1B in pSer2 and T4E pulldown samples.

(**A**) Native-PAGE EMSA of 26 repeat yeast CTD treated with Dyrk1a (left band) and untreated 26x yCTD (right band) and 26X T4E CTD (left band) compared to 26x yCTD (right band) before initiating pulldown experiment with CTD as the bait protein. (**B**) Spectral counts of proteins identified in the pSer2 sample compared to the control sample (unphosphorylated). (**C**) Spectral counts of T4E sample compared to the unphosphorylated control. (**D**) STRING physical network of proteins (n=46) that are positively enriched in both IP samples grouped by similar function. In green are CID proteins, yellow is mRNA splicing (HAS-72163), and in red is RNA Pol II transcription termination (HAS-73856).

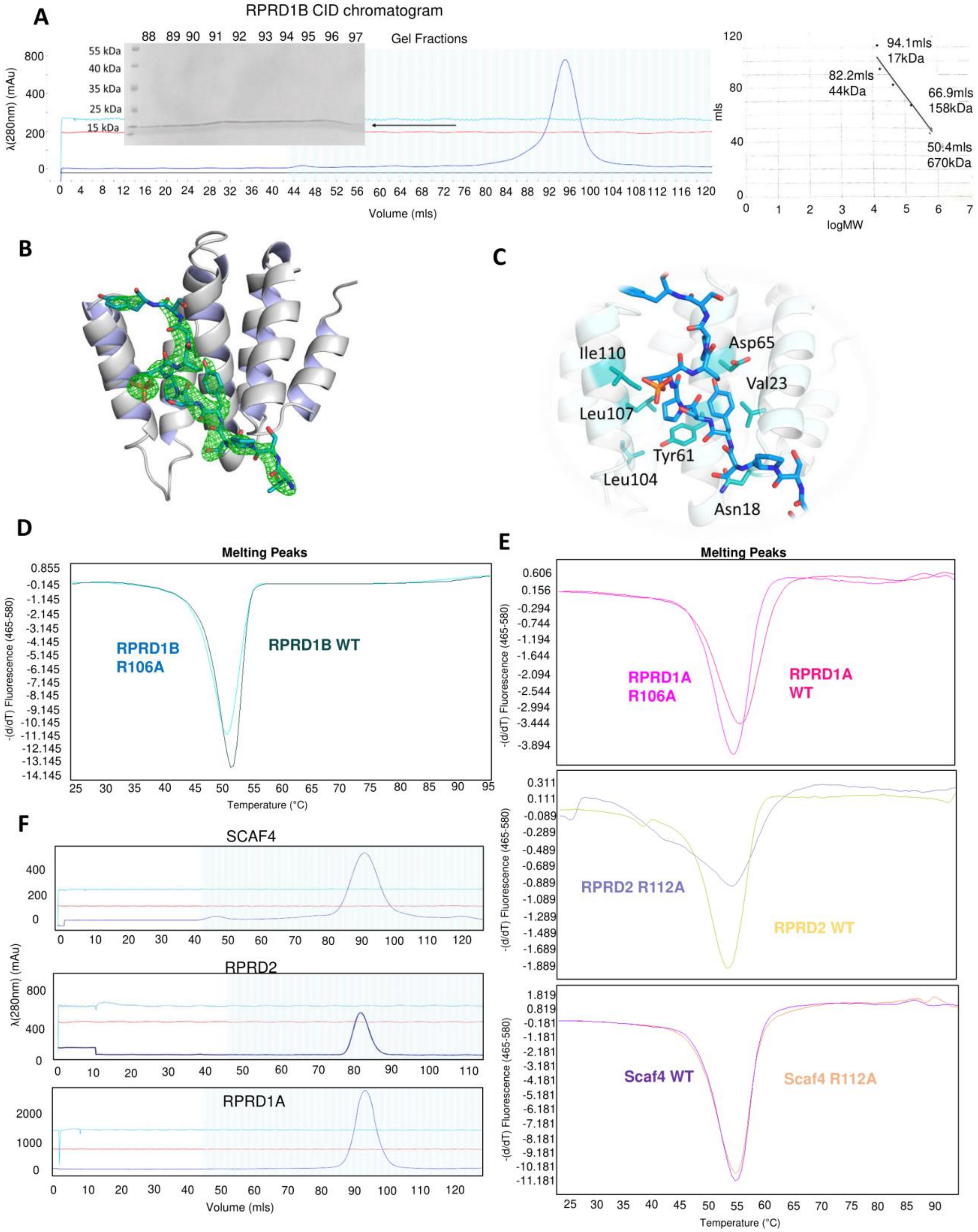

Fig. S4. Protein purification and characterization of CID proteins.

(**A**) SDS-PAGE of fractions isolated from size exclusion chromatography from RPRD1B-CID purification in *E. coli* and gel filtration chromatogram showing a single peak corresponding to a monomer of RPRD1B-CID. On the right hand side, is a standard curve plotting the MW of known proteins and their respective elution volumes. (**B**) Omit map of RPRD1B (light blue) with pThr4 CTD peptide (green density) contoured to 3σ. (**C**) Key backbone interactions shared between CID-containing proteins (**D**) Differential scanning fluorometry plot showing the melting temperature of the CID domain of RPRD1B and mutant. (**E**) Differential scanning fluorometry plots showing the melting temperature of the CID domain of RPRD1A, RPRD2, SCAF4 and pS2/pT4 binding mutants. (**F**) Size exclusion chromatography of purified SCAF4, RPRD1A, and RPRD2 CID protein Differential scanning fluorometry plot showing the melting temperature of SCAF4, RPRD1A, and RPRD2 and mutants.

.

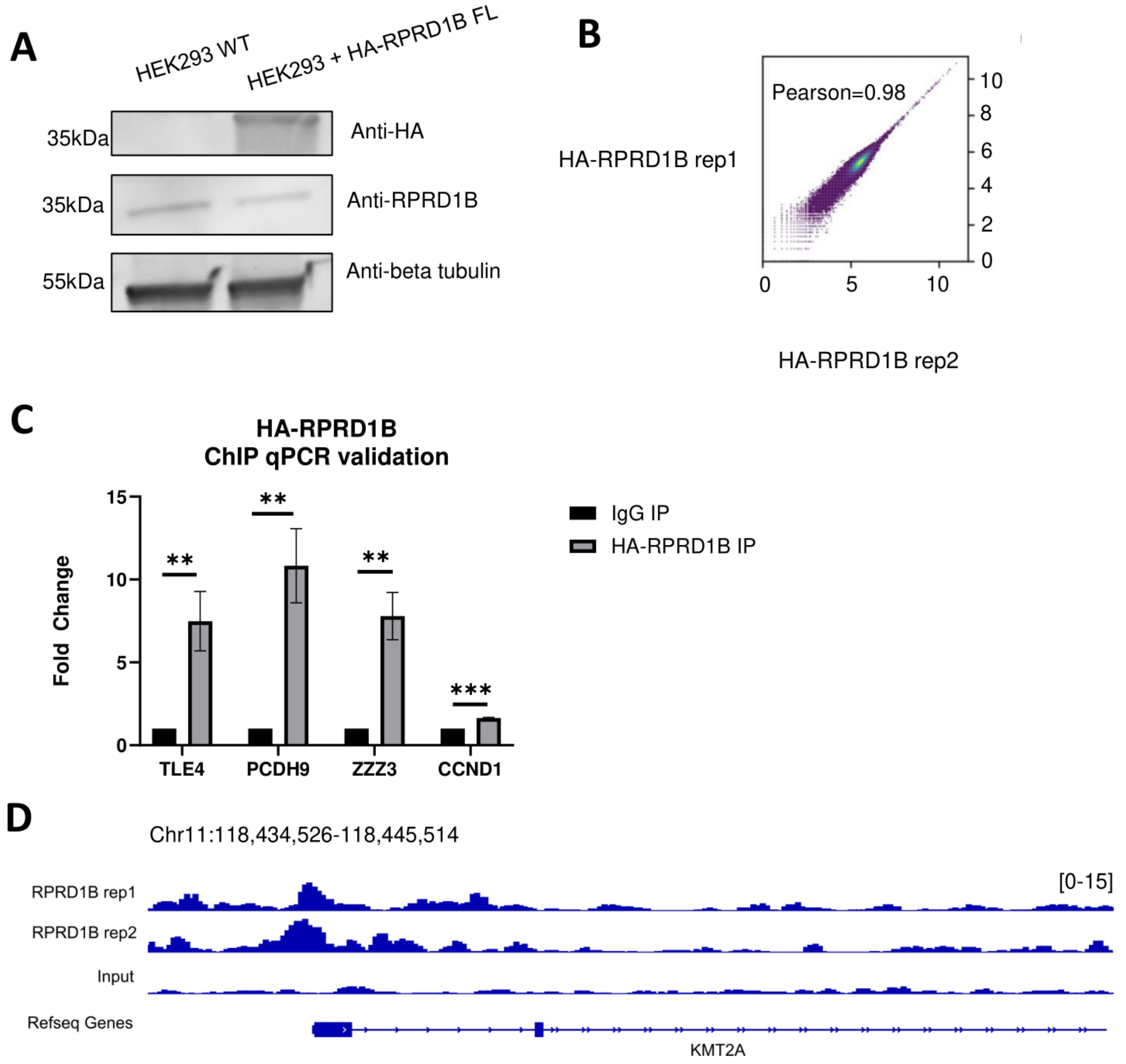

Fig. S5. Quality Control of RPRD1B ChIP-seq data.

(**A**) Expression of HA-RPRD1B in HEK293 cells (50 µg loaded) used for ChIP-seq. (**B**) Scatter plots showing the pearson correlation between ChIP-seq replicate datasets of HA-RPRD1B-bound regions. The genome was divided into bins of 10 kb and the number of mapped reads in the individual bins was calculated. (**C**) ChIP-qPCR analysis of peaks at promoter sites of selected genes from three biological replicates of HA-RPRD1B ChIP samples and IgG control samples. Fold enrichment was calculated by comparing the positive locus sequence in ChIP DNA over the negative IgG sample. For each data point, $n = 3$, error bars indicate standard deviation of three biological replicates. Comparison analysis was performed using unpaired t-

test. (**D**) IGV track example of RPRD1B replicates at a promoter genomic site. $p < 0.001$ (**) and $p < 0.0001$ (***).

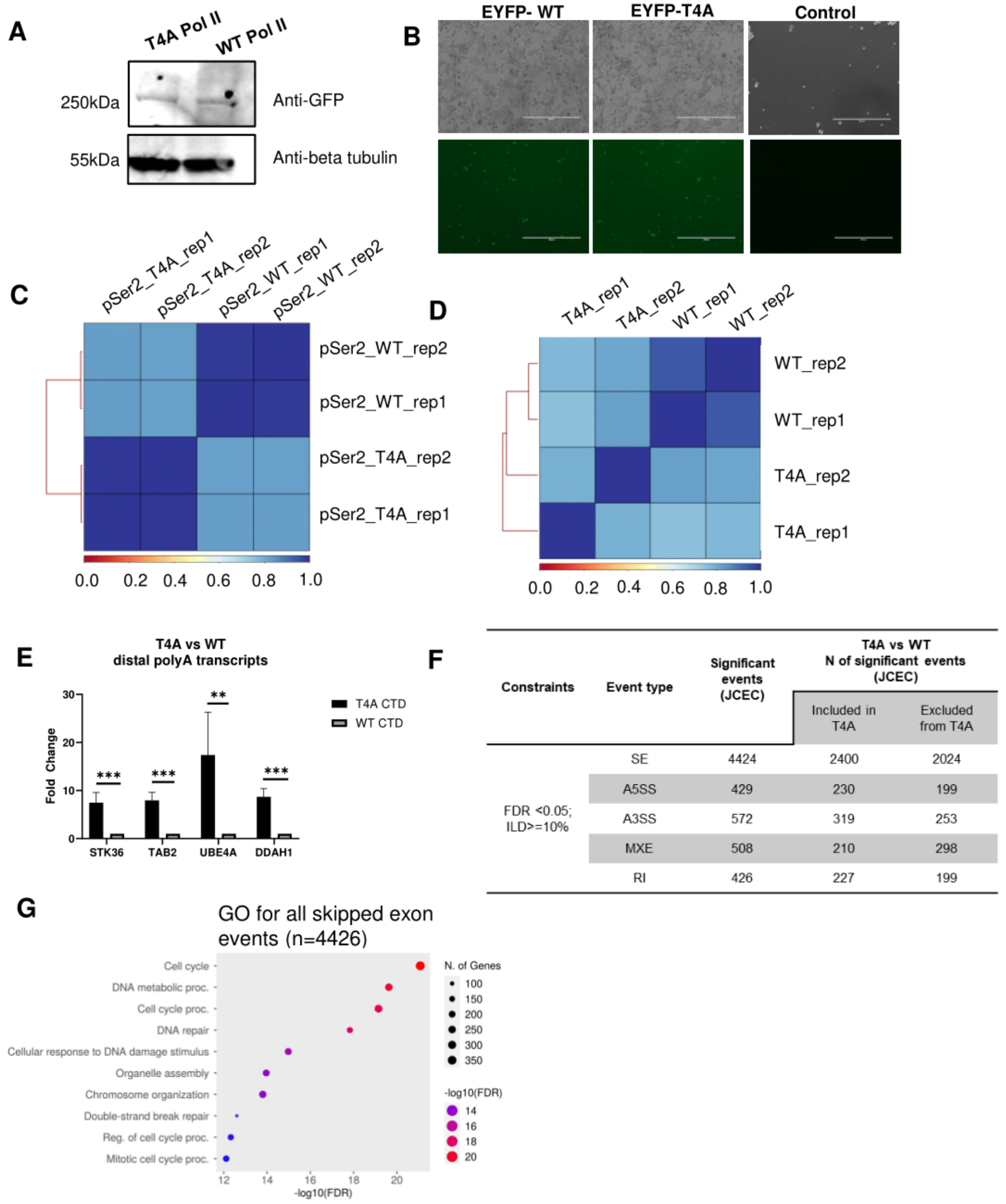

Constraints	Event type	Significant events (JCEC)	T4A vs WT N of significant events (JCEC)	
			Included in T4A	Excluded from T4A
FDR <0.05; ILD>=10%	SE	4424	2400	2024
	A5SS	429	230	199
	A3SS	572	319	253
	MXE	508	210	298
	RI	426	227	199

Fig. S6. Chip-seq and RNA-seq validation of T4A vs WT Pol II samples.

(**A**) Expression of alpha-amanitin resistant EYFP-WT and EYFP-T4A Pol II after 48 hours in HEK293 cells (50 μg loaded) used for RNA-seq. (**B**) Fluorescent and brightfield images showing

expression of EYFP-T4A and EYFP-WT after 48 hours with alpha amanitin (**C**) Pearson correlation analysis of pSer2 ChIP replicates under a T4A or WT RPB1 CTD background (**D**) Pearson correlation analysis of RNA-seq replicates for T4A and WT CTD samples. (**E**) Relative transcript expression of isoforms with different 3' ends after transfection of alpha-amanitin T4A or WT. For each data point, $n = 3$, error bars indicate standard deviation of three biological replicates. Comparison analysis was performed using unpaired t-test. (**F**) rMATS splicing analysis on mRNA-seq data from HEK293T cells expressing T4A or WT CTD. The table displays the defined five types of splicing events and the total number of significant events for each type (FDR <0.05 and ILD>=10%) (**G**) GO analysis of biological processes for transcripts with significant exon inclusion and exclusion events. $p < 0.001$ (**) and $p < 0.0001$ (***).

Table S1. pThr4 ChIP-seq peaks

Table S2. MS proteomic data

Table S3. Differentially expressed genes

Table S4. Alternative polyadenylation events

Table S5. Alternative splicing events

Table S6. Data collection and refinement statistics.

	RPRD1B + pThr4
**Data collection**	
Space group	C222
Cell dimensions	
a, b, c (Å)	66.32, 90.23, 135.07
α, β, γ (°)	90.00, 90.0, 90.00
Resolution (Å)	50.00-2.05 (2.09-2.05)*
Rsym/ Rpim	0.073(0.373)/0.061(0.260)
$CC_{½}$ ϒ	0.930 (0.886)
I / σ	15.2 (1.45)
Completeness (%)	92.1 (57.50)
Redundancy	3.9 (3.7)
**Refinement**	
Resolution (Å)	42.79-2.50 (2.59-2.50)
No. reflections	13183 (765)
R_{work}	0.2656 (0.3413)
$R_{free}^{\pm}$	0.3470 (0.4004)
**No. atoms**	2168
Protein	2037
Ligand/ion	93
Water	38
**B-factors (Å^2)**	
Protein	77.6
Ligand/ion	77.7
Water	82.2
**R.m.s. deviations**	
Bond lengths (Å)	0.009
Bond angles (°)	1.31
**Ramachandran plot**	
Favored	92.03%
Allowed	7.57%
Outliers	0.40^%
**Molprobity score**	2.24$^{\alpha}$ / 88th percentile$^{\beta}$

*Values for the corresponding parameters in the outermost shell in parenthesis.

ϒ$CC_{1/2}$ is the Pearson correlation coefficient for a random half of the data, the two numbers represent the lowest and highest resolution shell respectively.

$^{\pm}R_{free}$ is the R_{work} calculated for about 10% of the reflections randomly selected and omitted from refinement.

$^{\alpha}$The Molprobity score is calculated by combining the Clashscore with rotamer and Ramachandran percentage and scaled on the basis of X-ray resolution.

$^{\beta}$The 100th percentile is considered the best and the 0th percentile as the worst among structures of similar resolution.

^There is only one Ramachandran outlier, which corresponds to Lys 48 on chain B with strong electron density

Table S7. Sequence information of qPCR primers

QPCR primers	
**Gene**	**Sequence**
TLE4	FP: GCTTAGCTGTGACATCTCCGT<br>RP: CTGTGGCCTTCTTATCCCCA
PCDH9	FP: GTGTTGCTCCCAGTATCAT<br>RP: TCTGCCATGCAGTGGTAGC
INOC8	FP: CGACTGCGTACCTGCGCAAA<br>RP: ACTTCCACTCCCGGAATAGTC
ZZZ3	FP: CAGCCTCCCTGTCACGTGGAA<br>RP: GGAGCGCGCTCGCTCAGTA
ACTB	FP:TCAAGATCATTGCTCCTCCTG<br>RP:ACTCGTCATACTCCTGCTTG
DDAH1	FP: CCAGCCAATGTATGGTTAT<br>RP: TCACAGTACAACTCAACAC
STK36	FP: GAGGGATTATCCCTTAGC<br>RP: CAAGTATGGTGAGGCACAA
TAB2	FP: TGTTTGCAAGTGGCTGTG<br>RP: GCATCAAACGTAAGGGCT
UBE4A	FP: GCACATGCTCTAACTGCTCA<br>RP: AACAAACACTGCCAGGCA
CCND1	FP:AACTTGCACAGGGGTTGTGT<br>RP:GAGACCACGAGAAGGGGTGACTG
SMARCA4	FP: CAAAAGCCGAGCTGTGCAT<br>RP: ATTTCCCACAGGGACACGTT
SCAF1	FP: CTTCCTCAAACCTGACGAGC<br>RP: TTCCTGGTCTTGGATGGCTT
EP300	FP: ACA TGA ATC CAA TGC AGG CG<br>RP: AGGCATGGTGTTGTGGTTCA
